# Arctic plants can take up inorganic nitrogen year‐round

**DOI:** 10.1111/nph.71273

**Published:** 2026-05-18

**Authors:** Emil Alexander Sherman Andersen, Gesche Blume‐Werry, Chenxin Feng, Friederike Gehrmann, Niki Leblans, Anders Michelsen, Johan Olofsson, Josefine Walz, Ellen Dorrepaal

**Affiliations:** ^1^ Department of Ecology, Environment and Geoscience, Climate Impacts Research Centre Umeå University 981 07 Abisko Sweden; ^2^ Department of Ecology, Environment and Geoscience Umeå University 901 87 Umeå Sweden; ^3^ Faculty of Environmental and Agricultural Sciences Agricultural University of Iceland Hvanneyri IS‐311 Borgarness Iceland; ^4^ Terrestrial Ecology Section, Department of Biology University of Copenhagen 2100 Copenhagen Ø Denmark

**Keywords:** ^15^N‐tracer method, nitrogen allocation, nitrogen cycle, plant‐microbe interaction, seasonal nutrient dynamics, whole‐year N‐budget, winter

## Abstract

- Arctic tundra experiences strong climatic seasonality, with cold and long winters, but effective insulation by snow might enable plants and microbes to remain active in winter.
- We investigated year‐round seasonality of plant and microbial inorganic nitrogen (N) uptake, within‐plant allocation, and their dependence on snow depth in low‐Arctic tundra, using monthly *in situ* ^15^N‐pulse‐labelling.
- Plants and microbes took up the ^15^N‐label throughout the year, likely due to sufficiently deep and early snow that stabilised soil temperatures *c.* 0°C in both study sites. Surprisingly, we found only a few indications for higher plant and microbial ^15^N‐uptake and (estimated) plant total inorganic N‐uptake during the growing season compared to the cold season (i.e. the non‐growing season). Instead, N‐acquisition strongly varied within the cold season, with clearly higher uptake during the cold deep‐winter (November–March) than the warmer spring‐winter (April–May/June).
- Taken together, our results suggest that 78–82% of the annual plant inorganic N‐uptake may take place during the long cold season and as much as 50–56% during deep‐winter. This demonstrates the importance of nutrient dynamics during the cold season in the Arctic and challenges the general assumption that arctic plants remain dormant during the winter.

## Graphical abstract

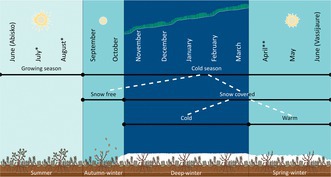

## Introduction

Arctic regions are characterised by long, cold, and dark winters that challenge plant growth and survival (Billings & Bliss, 1959; Shaver & Chapin III, 1980; Nadelhoffer *et al*., 1992; Walker *et al*., 1995). Processes vital for plant growth are therefore thought to be restricted to the short summer (Shaver & Chapin III, 1980; Nadelhoffer *et al*., 1992). Plant growth and biomass production in arctic tundra are further limited by low nutrient availability, especially nitrogen (N) (Wookey *et al*., 1995; McKane *et al*., 2002; LeBauer & Treseder, 2008; Zamin & Grogan, 2012; Zamin *et al*., 2014; Du *et al*., 2020). Acquisition and retention of N are thus of high priority for the generally perennial arctic plants. Arctic plant species can vary in their timing of (maximum) N‐uptake (McKane *et al*., 2002), and some species can take up N already just before snowmelt (Bilbrough *et al*., 2000), or in late autumn, even after leaf senescence (Blume‐Werry *et al*., 2019). However, the potential for plants to take up N also *during* the long Arctic winter has so far been overlooked.

**Fig. 1:**
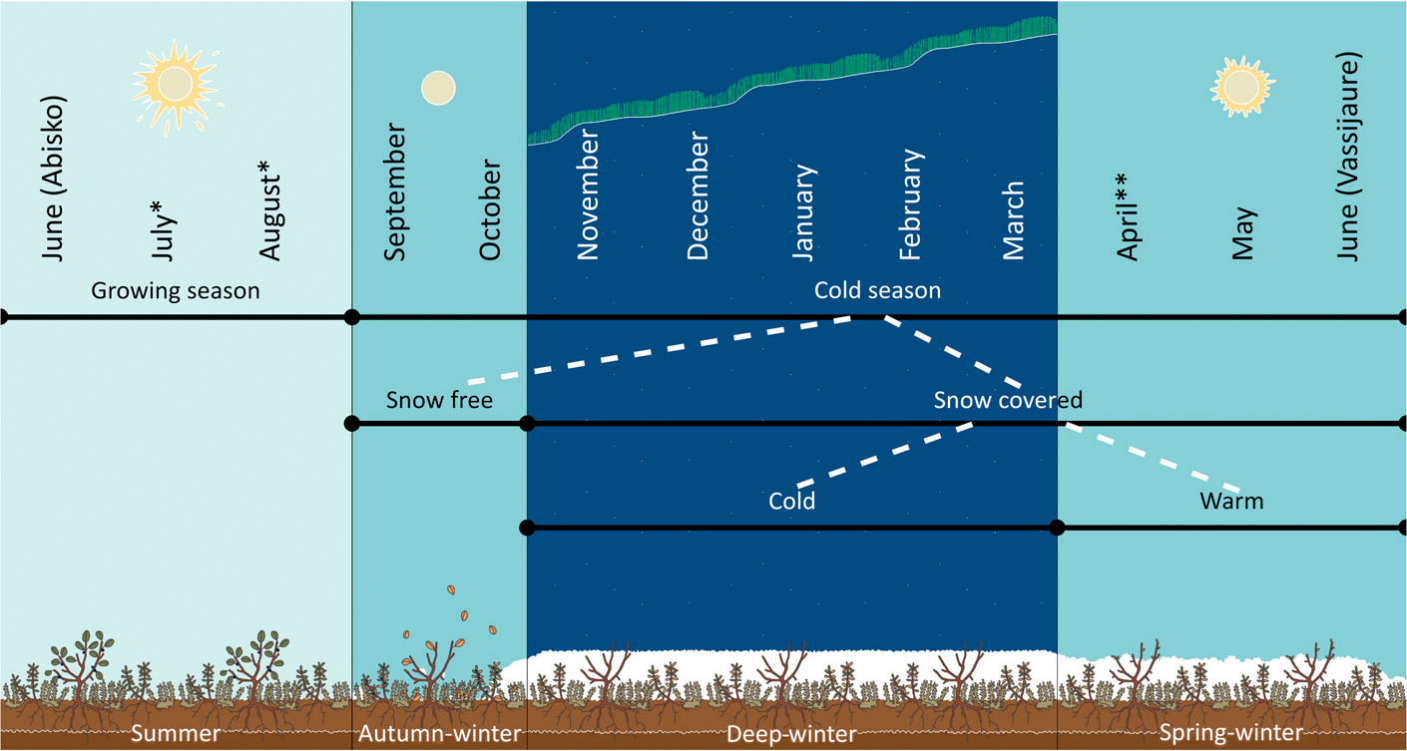
Schematic overview of year‐round, Arctic seasonal periods as used throughout the paper and their relation to the 15 repeated measuring periods (top) and the three *a priori* contrasts (middle) used for statistical testing of seasonality in N‐uptake. The ‘growing season’ or ‘summer’ refers to the aboveground productive season (Körner *et al*., 2023), during which most field research in the Arctic is traditionally performed. The ‘cold season’ (i.e. nongrowing season) starts with the peak of leaf senescence, strongly declining light availability, and first frost, and ends with the disappearance of snow cover (May in Abisko and June in Vassijaure). ‘Snow‐covered’ and ‘snow‐free’ parts of the cold season are distinguished by the first snow (November). The ‘cold’ and ‘warm’ parts of the snow‐covered period are separated based on returning light availability and more frequent above‐zero average air temperatures. The season names (bottom) for the three parts of the cold season are our own combinations of the names of the indigenous Northern Sámi seasons (Laptander *et al*., 2024) and the different stages of the cold season as defined by Olsson *et al*. (2003) and were chosen to not resemble traditional temperate seasons in order to avoid confusion about their ecological meaning in the Arctic. Background colours and drawings illustrate some typical seasonal changes in vegetation, snow cover, light and sky features. *Includes harvests in 2019 and 2020. **Includes harvests in early and late April 2020. Modified from drawings by Hélène Bocquet (Very Slow Studio), used with permission.

Winter‐like conditions, with frost and snow, may exist in much of the Arctic from September to May or June (Olsson *et al*., 2003). Yet, despite the generally adverse climate during the ‘cold season’ (i.e. the nongrowing season; see Fig. 1), the short‐statured tundra plants and their belowground roots may experience a much more benign microenvironment. Like 55% of the land in the Northern Hemisphere (Kreyling, 2010; IPCC, 2021), tundra is covered by snow during most of the cold season. Insulating snow can decouple soil from air temperatures, often stabilising soil temperatures *c*. 0°C (Olsson *et al*., 2003; Mörsdorf *et al*., 2019), depending on the soil temperature at the onset of snowfall, and snow depth and structure (Williams & Smith, 1989; Olsson *et al*., 2003). Dissolved minerals reduce the freezing temperature of soil pore water, improving access to water and nutrients (Williams & Smith, 1989). This subnival environment might therefore allow plants to remain active through the cold season.

Plant N‐uptake depends on the release of N in the soil, competition for N with other plants and soil microbes, and the intrinsic ability of the plant to take up nutrients at a given point in time. Several studies have suggested that up to half of the N used by arctic plants would need to be taken up outside the short growing season (Bilbrough *et al*., 2000; Grogan & Jonasson, 2003; Miller *et al*., 2009; Edwards & Jefferies, 2010; Larsen *et al*., 2012; Riley *et al*., 2021), as the net mineralisation during summer cannot supply their total N‐demand (Giblin *et al*., 1991; Jonasson *et al*., 1993; Schmidt *et al*., 2002). Microbial N‐mineralisation is strongly temperature‐limited (Giblin *et al*., 1991; Hobbie & Chapin, 1996; Mikan *et al*., 2002; Schimel *et al*., 2004; Brooks *et al*., 2011), contributing to seasonal variation in soil inorganic N availability. However, soil microorganisms can actively respire well below 0°C (Coxson & Parkinson, 1987; Panikov *et al*., 2006), and N‐mineralisation and immobilisation are highly dynamic just below 0°C (Buckeridge & Grogan, 2010). Substantial N‐mineralisation may therefore occur during deep‐winter and spring‐winter premelt (Hobbie & Chapin, 1996; Grogan & Jonasson, 2003; Kielland *et al*., 2006), especially under deeper snow (Brooks *et al*., 1996, 1999; Schimel *et al*., 2004). Given the length of the cold season, even a low N‐mineralisation rate could still constitute a substantial part of the inorganic N available annually for plant uptake.

Plant N‐uptake is an active process and is therefore both temperature‐ and energy‐dependent and expected to cease when winter approaches (Laine *et al*., 1994; Lloyd *et al*., 2011). Yet, when leaves senesce, arctic plant roots continue to grow for several more weeks (Iversen *et al*., 2015; Blume‐Werry *et al*., 2016; Sloan *et al*., 2016). Further, arctic plants can take up N even when energy supply from photosynthesis is limiting (Shaver *et al*., 1992) and plant carbon (C) allocation to belowground organs increases in late summer–early autumn (Olsrud & Christensen, 2004). Arctic plants are therefore capable of N‐uptake even in late autumn–early winter, when the soil has already started to freeze (Blume‐Werry *et al*., 2019). Equally high N‐uptake by plant roots in summer and winter in a temperate heath in Denmark (Andresen & Michelsen, 2005) further suggests that if soil temperatures are not too low in a relatively mild subnivean environment, plants might be able to continue to take up N even *during* ‘deep‐winter’ in the Arctic. Maximising their cold‐season N‐uptake might therefore be crucial to sustain growth of the generally N‐limited tundra plants, but it is currently unknown if and to what extent they utilise the soil N‐pools during the cold season.

Arctic plants are well adapted to conserve resources for survival in the harsh climate and nutrient‐poor conditions. Most tundra plants are perennial and have well‐developed storage organs such as stems, rhizomes and roots (Sonesson & Callaghan, 1991; Iversen *et al*., 2015). Further adaptations include the autumnal shedding of exposed aboveground tissues and efficient resorption of C and N into storage organs (Bret‐Harte *et al*., 2002; Olsrud & Christensen, 2004) for use in new growth during the next growing season. Well‐defended, longer‐lived leaves and woody shoots, on the other hand, help conserve nutrients in place and might extend the photosynthetic season. Allocation of nutrients may further depend on the sink strength of active tissues, which will vary with seasonal growth patterns. While allocation thus varies temporally, we know little about allocation *during* the cold season of the Arctic.

Our objective was to investigate the year‐round potential of plant inorganic N‐uptake and allocation, and its dependence on snow depth in arctic tundra. We tested the following hypotheses: (1) Plants and soil microbes take up inorganic N throughout the year, but the uptake is higher in summer than in the cold season. (2) Allocation of taken‐up N within plants varies through the year, with a larger proportion of N‐uptake stored belowground during the cold season than during summer. (3) A thicker snow cover increases and stabilises soil temperatures and thus promotes plant and microbial inorganic N acquisition during the cold season.

To test these hypotheses, we measured ^15^N‐uptake by both the plant and microbial communities, as well as its distribution among plant organs, over the course of a full year. We injected an inorganic ^15^N‐pulse label every month in the field, each time on different patches of the large plots, and harvested plants and soil after 3 wk. We further measured the size and isotopic composition of the dissolved soil N pool at each harvest and used these measurements to estimate total (^15^N + ^14^N) plant inorganic N‐uptake. We conducted the study at two low‐Arctic mixed heath sites with different snow‐cover depth and duration.

## Materials and Methods

### Sites

Our sites were located in the Torneträsk area, northern Sweden (Supporting Information Fig. S1). A strong precipitation gradient in the area allowed us to select two low‐Arctic sites with contrasting snow conditions. One site (*c*. 405 m above sea level (asl), 68°21′N, 18°49′ E) was close to the Abisko Scientific Research Station, with annual precipitation of 347 mm and mean annual temperature of 0.3°C (SMHI, 1991–2020). The second site (*c*. 610 m asl, 68°25′N, 18°15′E) was 30 km further west near Vassijaure, with annual precipitation of 859 mm and mean annual temperature of −0.5°C (SMHI, 1991–2020, Katterjåkk weather station *c*. 3 km away). A substantial portion of the precipitation falls as snow (Kohler *et al*., 2006), resulting in greater snow depth in Vassijaure. The vegetation at both sites is mixed tundra heath, with evergreen and deciduous shrubs as well as graminoids. Dominant vascular plant species include *Empetrum hermaphroditum* Hagerup, *Betula nana* L., *Vaccinium uliginosum* L., *Andromeda polifolia* L., and *Carex* L. spp. (see Fig. S1; Table S1). The soil is shallow (< 1 m), with a 5–10‐cm‐thick organic horizon, underlain by till in Abisko and bedrock (quartzite, metagreywacke) in Vassijaure (SGU, 2010).

### Experimental design

The experiment took place from summer 2019 to summer 2020. In June 2019, five plot areas were designated at each site (i.e. *n* = 5), with the shortest distance 7 m apart. The plot areas did not have a strict size or shape but were large enough so that we could select 17 separate patches within each plot, of which 15 were used for single ^15^N‐pulse labelling in sequential measuring periods (MPs), every 4 wk throughout a full year, and two were used for unlabelled control samplings (see ‘Application of ^15^N‐label’ section). To overcome spatial heterogeneity of Arctic soils and vegetation and to prevent this from biasing comparison of ^15^N‐recovery of different patches through time, all patches (circular, diameter *c*. 10 cm) were selected based on a representative vegetation cover (Table S1), and the requirement to contain the shoot base of at least one evergreen shrub, one deciduous shrub and one graminoid; usually *E. hermaphroditum*, *A. polifolia*, *V. uliginosum*, and *Carex* spp. By including a mix of different, common functional groups, we accounted for potential differences in the seasonality of their ^15^N‐uptake – caused by, for example, differences in the timing of leaf senescence – in order to maximise the chances to detect winter ^15^N‐uptake of the whole vegetation if any of these groups was capable of winter N‐uptake. The patches were further selected to have, where possible, absent or low moss cover to minimise potential loss and spread of ^15^N‐label aboveground due to strong absorption of liquids by moss. Patches within plots were selected at least 1 m apart to reduce the risk of lateral ^15^N‐contamination between patches labelled during different measurement periods. Finally, we aimed for a minimum soil depth of at least 10 cm, which was not always possible in Vassijaure due to near‐surface stones or bedrock.

All patches were trenched once in late June/early July 2019 during plot establishment by cutting a circle of 24 cm diameter down to 10 cm soil depth around the core patch to confine root ^15^N‐uptake to the plants with roots and shoots inside the patch and to restrict uptake by plants with most of their biomass outside the patch. To minimise disturbance to plants, microbes and soil hydrology, trenches were placed at 7 cm from the patch edges, and no barriers were installed in the trenches. All patches were marked with coloured sticks defining the edges (Fig. S1), and their positions were marked with dGPS to facilitate localisation during both snow‐free and snow‐covered periods. The sequence of ^15^N‐labelling from July 2019 to August 2020 across patches was stratified to avoid contamination of other patches and minimise disturbance of the snowpack: patches were labelled from downhill first and in winter from the periphery inwards.

### Environmental conditions

Comparing sites along a natural environmental gradient, as we do here, has the advantage that plants and soil communities are adapted to long‐term differences in conditions but may come with co‐varying conditions (e.g. Dunne *et al*., 2004). We therefore characterised several climatic conditions during our measurement year. Air temperatures were obtained from the meteorological stations in Abisko and Katterjåkk (SMHI), at 1 and 3 km from the sites in Abisko and Vassijaure, respectively. Further, soil temperature and moisture (volumetric water content) at 5 cm depth were logged in each plot at both sites every hour (5TM via EM50 data loggers; Decagon Devices Ltd, Pullman, WA, USA). Soil subsamples from the harvests (see ‘Plant and soil harvest and processing’ section) were also used to determine soil moisture gravimetrically and convert it to volumetric water content using soil bulk density. Snow depth was measured at *c*. 10 points at the periphery of each plot during ^15^N‐labelling days, but during snowmelt (May–June), also at the plant and soil harvest days.

### Application of ^15^N‐label

Repeated cycles of ^15^N‐injection plus harvest were performed throughout a full year to assess seasonal variation in the potential uptake of inorganic N. Each cycle consisted of 3 wk between injection and harvest, and 1 wk between harvest and the next injection of a different patch (i.e. 4 wk between injections). Three weeks provide sufficient time for detecting ^15^N‐uptake (Andresen *et al*., 2008).

We used dual‐labelled ^15^N ammonium‐nitrate (^15^NH_4_^15^NO_3_; 98 atom%) for pulse labelling of vegetation patches to ensure that we would be able to register ^15^N‐recovery irrespective of whether the species in our plots at the time of our measurement would have a preference for ammonium or nitrate (McKane *et al*., 2002; Nordin *et al*., 2004). For each 3‐wk MP, we injected 19 ml of 1.9 mM double‐labelled ^15^N‐solution to a different patch in each plot: 1.084 mg ^15^N per patch, equivalent to 136.67 mg N m^−2^, that is, a nonfertilising level (Templer *et al*., 2012). To evenly distribute the ^15^N‐label over the patch, a 10 cm × 10 cm acrylic plate with 19 injection points (openings) distributed triangularly to form a hexagonal shape (2 cm between injection points, max. diameter of hexagon 8 cm) was laid over the patch, and 10‐cm long metal pins were pushed 7 cm into the soil through each opening. Then, one by one, the metal pins were pulled out, and 1 ml of ^15^N‐solution was injected immediately into each hole underneath to a soil depth of 7 cm. The distance between injection points was based on the intention to spread the label as homogeneously as possible through the soil of the patch while keeping disturbance of the soil and plant roots to a minimum, and earlier try‐outs of injection with black ink during winter outside the plots, which indicated 1 cm of radial spread of injected liquid. The volume of injection liquid was equal to the volume of the 7‐cm deep injection hole in the soil. In this way, we made sure that the ^15^N‐label was within reach of plant roots throughout the whole patch, even if there may have been small‐scale heterogeneity in the distribution of the roots or of soil structure within the patches. Injection of this small amount (19 ml in total per patch) of nonfrozen solution had only a very small and short‐lasting impact on the soil temperature of the patch in winter (max. 0.44 ± 0.08°C (0.92 ± 0.25°C) temperature increase in Abisko (Vassijaure, respectively), 34 min (7–74 min) (19 min (15–119 min)) after injection; 0.15 ± 0.05°C (0.16 ± 0.16°C) after 3 h; tested on 9 (11) March 2020 (mean ± SE for temperature increases; median (interquartile range) for time intervals; *n* = 5 patches per site)). To prevent the spread of label to aboveground plant tissues in subsequent patches, the labelling equipment was thoroughly cleaned between patches with water (in summer 2019) or ethanol (in the cold season and summer 2020) to avoid clogging by ice. During the snow‐covered period, snow was shovelled and carefully brushed from the target patch just before labelling and re‐applied directly afterwards.

### Plant and soil harvests and processing

Three weeks after the application of ^15^N‐label, the vegetation and soil of each labelled patch were harvested with a soil corer (10 cm diameter PVC corer in summer, 11 cm metal corer in winter) exactly around the injection hexagon to a depth of 10 cm, wherever possible, given solid ice and soil rock conditions. After harvesting, the soil cores were brought to the lab and, if necessary, left to thaw at 4°C before processing within 48 h of the harvest (or within 24 h if not frozen).

The natural ^15^N‐abundance of plants, microbial biomass, and soil extracts (see ‘Recovery of ^15^N’ section) was determined from an average of two control harvests at each site, collected at the same time as the labelled samples in September 2019 and March 2020. We used only two time points for our control harvests, as any natural variation in ^15^N throughout the year would be insignificant compared to the highly enriched ^15^N‐labelling (Larsen *et al*., 2012).

For each harvested core, litter and bryophytes were discarded, and vascular plants were carefully removed, maintaining roots attached as far as possible. The vascular plants were then partitioned into aboveground parts, coarse roots (CR, > 2 mm diameter), and fine roots (FR, < 2 mm diameter). The remainder of the soil core was weighed, homogenised, and a representative subsample of *c*. 40 g was carefully root‐sorted for 1 h to estimate bulk root biomass. Subsamples were weighed before and after sorting: The weight before sorting was used to scale the bulk root biomass (fine and coarse) to the whole core, whereas the weight after sorting was used to calculate root‐sorted soil proportion for inorganic N and microbial N soil extractions (see ‘Recovery of ^15^N’ section). Each plant part was rinsed three times in water and three times in a 0.5 mM CaCl_2_ solution to rinse off any ^15^N potentially attached to its surface (Persson *et al*., 2003). All plant samples were dried at 70°C until constant mass before being weighed, ground by ball milling (Retsch MM301, Retsch GmbH, Haan, Germany), and analysed for ^15^N (Flash EA 2000 coupled to a DeltaV IRMS, Thermo Fisher Scientific, Bremen, Germany; analysed by SLU Stable Isotope Lab, Umeå, Sweden). All ^15^N‐analyses were calibrated with international standards IAEA‐600, IAEA‐N‐2, USGS40, USGS41, and N‐concentrations were calibrated with standards of atropine and NIST 1515 apple leaves.

For analyses of inorganic N availability and microbial N, two aliquots of 6 g of the root‐sorted soil were extracted with 30 ml MilliQ demineralised water. One aliquot was fumigated with chloroform for 72 h (adjusted from Hobbie, 1998) before extraction to obtain microbial N. The extractions were shaken for 2 h at 150 rpm (2.5 Hz) and filtered (Ahlström‐Munksjö Munktell quantitative filter paper, pore size 1–2 μm). The remaining soil was washed with 10 ml demineralised water, filtered, and added to the first part of the extract. We used demineralised water for extraction to avoid complications with salt precipitate during freeze‐drying before isotopic analyses, which provides a realistic estimate of N availability in the natural environment (Nordin *et al*., 2004; Halbritter *et al*., 2020). Furthermore, water and salt extracts (such as KCl) yield almost identical N‐concentrations in organic tundra soils (Clemmensen *et al*., 2008).

The filtered extracts (fumigated and nonfumigated) were frozen until analysis for inorganic N (NH_4_^+^ and NO_3_^−^; QuAAtro39 Continuous Segmented Flow Analyzer, SEAL analytical, Norderstedt, Germany), after which the remaining extracts (between 5 and 37 ml) were freeze‐dried onto Whatman quartz microfiber filters (grade QM‐A) and analysed for total ^15^N (Isoprime IRMS; Elementar, Cheadle Hulme, UK coupled to a Eurovector EA, Pavia, Italy). Total dissolved nitrogen (TDN) concentrations for fumigated and nonfumigated samples were also determined by the IRMS, except for the extracts from March and August 2020 (nonfumigated) and June 2020 (fumigated), which, for technical reasons, were analysed by TOC/TN Analyzer (Formacs^HT‐I^ TOC/TN Analyzer, Skalar Analytical B.V., Breda, the Netherlands). Microbial nitrogen (MBN) concentration was calculated as the difference between the TDN concentration of the fumigated and nonfumigated extracts. The MBN was corrected by an extractability factor (K_EN_) of 0.4 to account for nonchloroform extractable N (Jonasson *et al*., 1996; Schmidt *et al*., 2002; Clemmensen *et al*., 2008). Concentration and atom% ^15^N of control (unlabelled) samples were averaged per site and per ecosystem component analysed: Plant organ (shoots, CR, and FR), MBN, TDN, and extracted inorganic N.

Finally, the gravimetric soil water content was determined from a separate subsample (30–40 g) of the nonroot‐sorted soil cores by removing roots and stones for 10 min, weighing, drying at 70°C for a minimum of 48 h, and re‐weighing.

### Recovery of ^15^N

Recovery of ^15^N‐label (% of added ^15^N) in plant organs, TDN and soil microbial N‐pool was calculated as detailed in Methods S1. To better understand the seasonal dynamics between plant–soil‐microbe N‐distributions and avoid conflating potential seasonal variation in loss or immobilisation of N as the underlying factor, we defined total ecosystem ^15^N‐recovery as the sum of plant, microbial and TDN ^15^N‐recovery (but excluding abiotically immobilised N), which reflects the ^15^N available to plants during the 3 wk between injection and harvest. For each pool – plant community, MBN, and TDN – we then analysed both the ^15^N‐recovery relative to total injected ^15^N, referred to as *absolute* recovery, and the ^15^N‐recovery relative to total ecosystem recovery, referred to as *relative* recovery. Plant ^15^N‐recovery was analysed both for the whole plant community and partitioned into different organs (shoots, CR and FR). For the different organs, recovery was expressed as a percentage of total plant recovery.

### Estimated plant total inorganic N‐uptake

We estimated year‐round variation in plant total inorganic N‐uptake as the sum of absolute ^15^N‐recovery and estimated uptake of ^14^N to account for potential seasonal changes in soil inorganic N‐pools and turnover, which would dilute the ^15^N‐label differently and thereby potentially confound the seasonal signal of ^15^N‐recovery alone. The plant total N‐uptake was estimated based on the measured ^15^N‐recovery and the estimated average ^15^N isotopic ratio (^15^R=^15^N : ^14^N) in the dissolved inorganic N‐pool during the 3‐wk labelling period. We assumed that the uptake of each isotope was proportional to their average ratio in the soil inorganic N‐pool and used ^15^R^−1^ to estimate how much ^14^N was taken up per ^15^N recovered, and thus the plant ^14^N‐uptake (see Methods S1). Natural ecosystems and soils are inherently heterogeneous, and multiple processes can happen simultaneously and cannot be controlled for as stringently as in laboratory experiments, which may challenge some assumptions underlying the isotope pool dilution method. We therefore homogenised the target vegetation during our selection of the patches and optimised the application of the label to overcome spatial heterogeneity in vegetation, soil and label (see ‘Experimental design’ section). Further, we performed a sensitivity analysis (see Methods S1) to analyse how the assumed distribution of the remaining ^15^N‐label over the different parts of the extractable N‐pool (inorganic‐N or organic‐N) at harvest would affect our total N‐uptake estimations, which turned out almost identical (Table S2), suggesting low sensitivity of our estimations to the assumed distribution. Still, as the ^14^N‐uptake was not measured directly, we consistently refer to the total inorganic N‐uptake as *estimated* total plant inorganic N‐uptake.

### Data analysis

Total ecosystem ^15^N‐recovery; plant community, MBN and TDN ^15^N‐recovery; plant ^15^N‐allocation; estimated plant total inorganic N‐uptake; and snow‐cover were analysed by repeated measures analysis of variance (rmANOVA) using linear mixed‐effect models with R v.4.2.3 (R Core Team, 2023) and package nlme (Pinheiro *et al*., 2023) and ANOVA from the package car (Fox & Weisberg, 2019), to test the effect of time (MP; i.e. the 15 sequential labelling cycles on different patches) and snow regime (site), and in the case of plant ^15^N allocation, organ (shoots vs roots and CR vs FR). MP and site (and organ) as well as their interactions were set as fixed effects. Plots (1–5) nested within site (Abisko, Vassijaure) were included as random effect to account for the repeated measures (patches) within the plots.

For further testing differences in ^15^N‐recovery and estimated plant total inorganic N‐uptake between the MPs in specific seasons or parts of seasons, we reduced the large number of possible pairwise comparisons by defining *a priori* orthogonal contrasts (Fig. 1). The first contrast, testing parts of each of our hypotheses, was between MPs in the ‘growing season’ and the ‘cold season’. Then, because of the long duration and variation in conditions of the cold season, we analysed variation between different parts of the cold season, that is contrast 2 between MPs in the ‘snow‐covered’ and the ‘snow‐free’ parts within the cold season, and contrast 3 between MPs in the ‘cold’ vs the ‘warm’ parts of the snow‐covered season. Contrasts were defined and performed per site (irrespective of whether we found a site × time interaction first), as the end of the snow‐covered period, and thus also the cold season, differed between sites (May in Abisko, June in Vassijaure).

Normality and homoscedasticity were visually inspected with QQ plot and fitted vs residual plots. Variables were subsequently arcsin‐transformed to account for percentage data, except for plant recovery and TDN, which were log(*x* + 1) transformed.

## Results

### Environmental seasonality

**Fig. 2:**
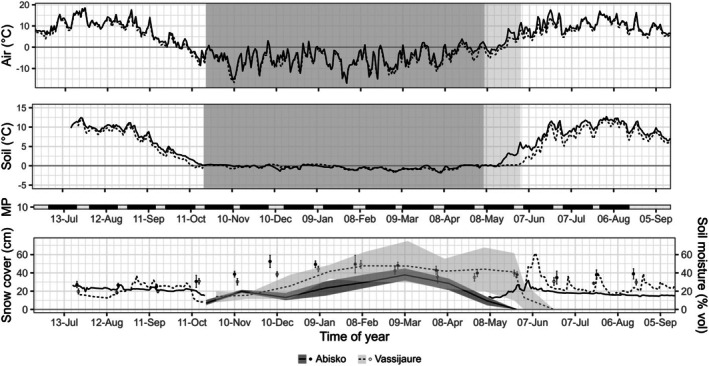
Environmental data from the experimental locations over the duration of the experiment. Air (top panel) and soil (mid panel) temperatures in Abisko (solid lines) and Vassijaure (dashed lines) from July 2019 to August 2020. Air temperatures from the closest SMHI meteorological stations for each site: Abisko and Katterjåkk (*c*. 1 and 3 km from the Abisko and Vassijaure sites, respectively). Soil temperatures were measured with five sensors per site, one in each plot. Temperatures were averaged over each diel (24‐h cycle). The grey‐shaded areas in the top and mid panels indicate the snow‐covered periods, which ended *c*. 1 month earlier in Abisko than in Vassijaure. The snow‐covered period was defined as the first measured snow on the plots to the last measuring period (MP) where snow was found. MP (thin mid panel): solid black indicates the 15 measurement periods (3 wk each), from ^15^N‐label injection to harvest; white indicates the periods (*c*. 1 wk each) between harvest and labelling of the next patch of each plot. Snow cover depth (bottom panel, left axis; solid (Abisko) and dashed (Vassijaure) lines with grey shading indicating min. and max. range) and soil moisture content (bottom panel, right axis; % vol: dashed and solid lines during the snow‐free period; and gravimetric water content (GWC): solid (Abisko) and open (Vassijaure) dots). Snow depth was measured as an average of 10 points on the periphery of each plot for five plots per site at the ^15^N‐labelling time point, except for spring, when snow cover was measured at both ^15^N‐labelling and harvest. Volumetric soil moisture was measured with five sensors per site, one in each plot, and averaged over each diel (24 h) cycle, and is not shown for the snow‐covered period, as it is difficult to measure continuously in frozen soil. Soil GWC was measured from each harvested core (5 plots per site) and converted to volumetric water content with soil bulk density. Error bars represent ±SE. Note that the environmental monitoring at the sites only started shortly after the first injection of the label had taken place.

The two sites had contrasting snow cover over time (sites, MP, site × MP, all *P* < 0.001). For both sites, the first snow cover was registered around the same time (Abisko 21 October 2019 and Vassijaure 28 October 2019), but the snow was deeper in Vassijaure than in Abisko from approximately mid‐December through the rest of the cold season. The snow continued to be deep in Vassijaure until May (40.9 ± 9.7 cm, with three out of five plots > 50 cm snow, Fig. 2), when Abisko was almost snow‐free (9.4 ± 1.6 cm). As a consequence, the snow‐covered period was 1 month shorter in Abisko than in Vassijaure (spring melt‐out 27 May and 22 June 2020, respectively). The maximum average snow depth in both sites exceeded 30 cm in February but was on average 24 cm deeper in Vassijaure than in Abisko, a difference that increased to 54 cm by May (Fig. 2). The diurnal average soil temperatures at 5 cm depth stabilised in both sites at 0°C to −2°C (absolute min. values −3.4°C in Abisko in December and −4.1°C in Vassijaure in March and April), whereas diurnal average air temperatures above the snow fluctuated between 0°C and −15°C during the snow‐covered season (absolute minimum values −26.0°C in Abisko and −23.3°C in Vassijaure in February) (Fig. 2).

### Seasonality of total ecosystem ^15^N‐recovery

**Fig. 3:**
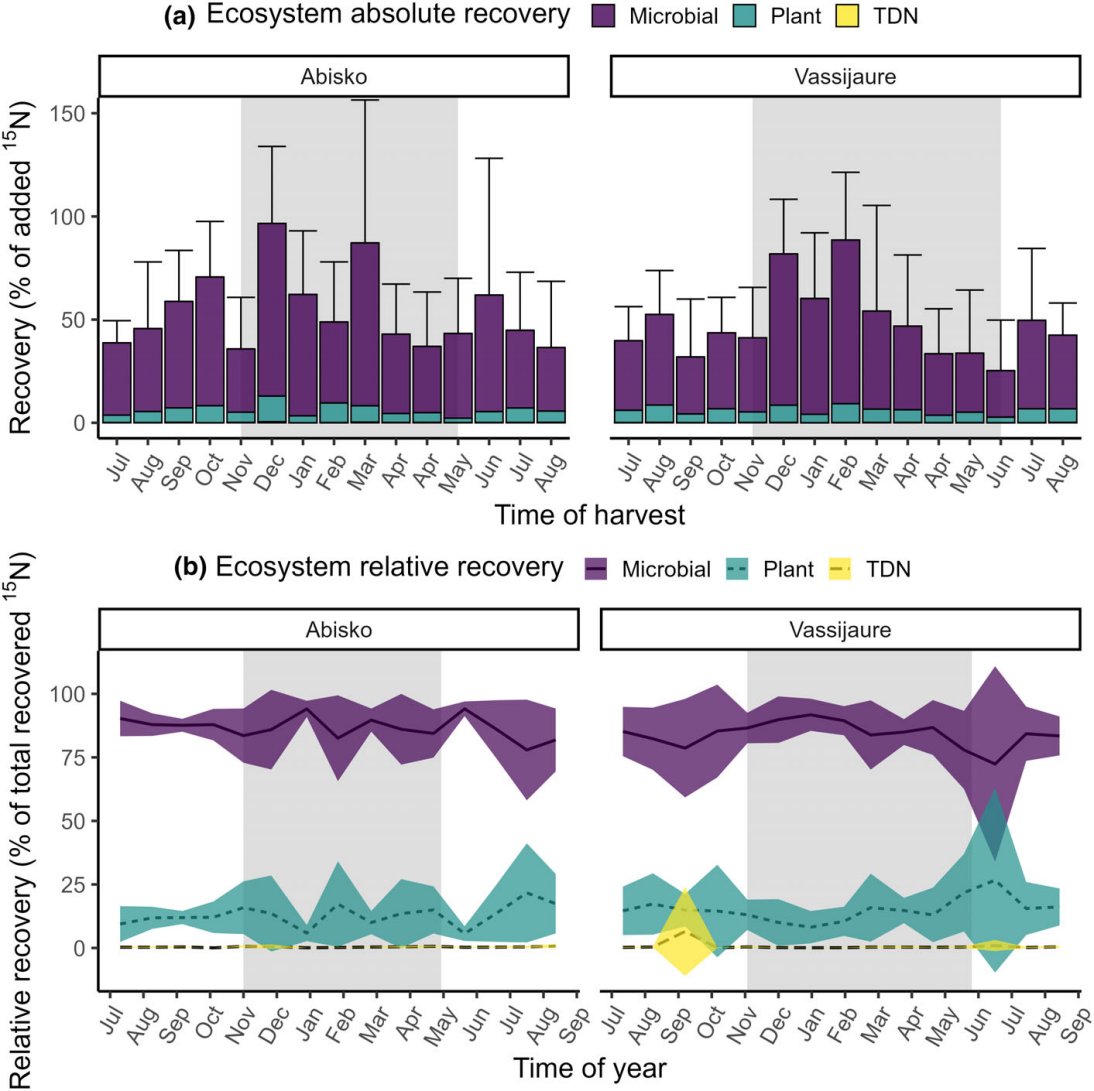
Year‐round seasonal patterns of (a) total ecosystem recovery of ^15^N tracer (% of label addition) and its distribution over plant and microbial communities and TDN (total dissolved nitrogen); and (b) the relative distribution of recovered ^15^N tracer (% of the total ecosystem recovery) in each of three ecosystem components: microbial, plant communities and TDN, in Abisko (left) and Vassijaure (right), 2019–2020. Note that in (b), for each component, the relative recovery is plotted with respect to zero, that is the lines are not stacked. The high relative TDN recovery in Vassijaure (September) is from one outlier plot where, at this time point only, total ecosystem recovery was low (< 0.2%), but absolute TDN recovery (0.055%) was not higher than the other replicates (see Supporting Information Fig. S5). Months refer to when the experimental plots were harvested and represent recovery over the preceding 3‐wk period between labelling and harvest. Also note that there is an early April and a late April measurement in (a), where each bar is labelled on the *x*‐axis by the harvest month, whereas in (b) the *x*‐axis is a continuous timeline and the results for each harvest are plotted at the exact harvest date. The grey shading in the background indicates the snow‐covered period. Error bars and shading around lines represent 95% confidence intervals (*n* = 5). The error bars in (a) are for total ecosystem recovery (see Figs S2, S3 and S5 for the values and error bars for plants, microbes and TDN, respectively).

Total ecosystem recovery of ^15^N‐label did not differ significantly between the two sites nor interacted between sites and MPs, but varied across the year (MP: χ^2^ = 50.1, df = 14, *P* < 0.001, Fig. 3a). Total ecosystem recovery for MPs in the growing season and in the cold season was not different from each other (contrast 1) nor in the snow‐free vs the snow‐covered parts of the cold season (contrast 2). Instead, total ecosystem recovery was higher for MPs in the cold part (deep‐winter) than in the warm part (spring‐winter) of the snow‐covered period (Abisko: 66 ± 7.8% recovery November–March vs 41 ± 5.0% April–May; *t* = 2.6, df = 56, *P* = 0.012; Vassijaure: 65 ± 6.2% November–March vs 35 ± 5.0% April–June; *t* = 4.8, df = 56, *P* < 0.001) (contrast 3).

### Seasonality of whole‐plant and microbial ^15^N‐recovery

Absolute plant ^15^N‐recovery varied across the year (MP: χ^2^ = 27.7, df = 14, *P* = 0.016) but not between sites, nor did sites and MPs interact (Figs 3a, S2), but when each site was tested separately, MPs only varied significantly in Abisko (χ^2^ = 33.7, df = 14, *P* = 0.002). This difference in absolute plant recovery in Abisko was not between MPs during the growing season and in the cold season, but within the cold season: recovery was only 0.81 times as high for MPs in the snow‐free (6.2 ± 1.0%) as in the snow‐covered part of the cold season (7.6 ± 1.0%; t = −2.2, df = 56, *P* = 0.031), and more than twice as high for MPs during the cold (deep‐winter: 7.6 ± 1.5%) as during the warm part of the snow‐covered period (spring‐winter: 3.7 ± 0.6%; *t* = 3.0, df = 56, *P* = 0.004).

Absolute microbial ^15^N‐recovery also varied across the year (MP: χ^2^ = 39.8, df = 14, *P* < 0.001), but not between sites, nor did sites and time of year interact (Figs 3a, S3). There were no differences between MPs in the growing season vs the cold season, or in the snow‐free vs the snow‐covered parts of the cold season, but absolute microbial recovery was higher for MPs during the cold part of the snow‐covered season (deep‐winter: December–March, Abisko 58.2 ± 7.2%, Vassijaure 58.4 ± 5.9%) than during spring‐winter (April–May(June), Abisko 37.2 ± 4.9%, t = 2.1, df = 56, *P* = 0.037; Vassijaure 30.4 ± 4.8%, t = 3.9, df = 56, *P* < 0.001).

Of the total ecosystem‐recovered ^15^N, 72.4–94.1% was found in soil microbes. The relative recovery in plants and microbes (as the percentage of total ecosystem‐recovered ^15^N) did not significantly vary across the year or between sites, nor was there a significant interaction (Fig. 3b).

### Seasonality of ^15^N‐allocation within plants

**Fig. 4:**
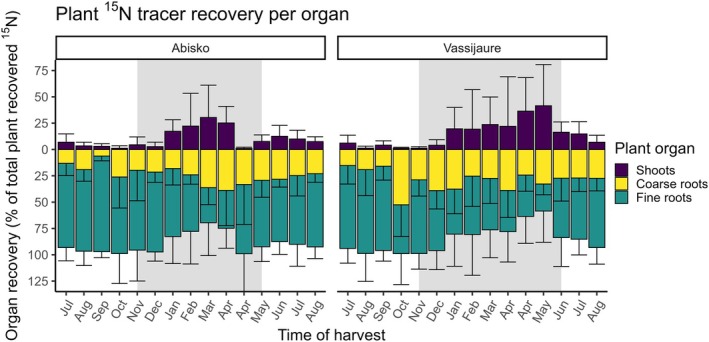
Year‐round seasonal patterns of proportional plant allocation of recovered ^15^N tracer between different organs relative to the total recovery in the plant community in Abisko (left) and Vassijaure (right), 2019–2020. The plant recovery is split into aboveground (shoots) and belowground (coarse roots > 2 mm, and fine roots < 2 mm), and bars are stacked and add up to 100%. Note that there is an early April and a late April measurement. The grey shading in the background indicates the snow‐covered period. Monthly bars refer to when the experimental plots were harvested and represent estimated uptake over the preceding 3‐wk period between labelling and harvest. Error bars represent 95% confidence intervals (*n* = 5).

Only a small fraction of the ^15^N in plants was recovered in the aboveground biomass, whereas the majority stayed belowground (Fig. 4; organ: χ^2^ = 670.0, df = 2, *P* < 0.001), primarily in the FR and less in the CR, reflecting that more than 80% of the plant biomass was located belowground (Fig. S4). These differences were slightly stronger in Abisko than in Vassijaure (site × organ: χ^2^ = 16.2, df = 2, *P* < 0.001). The allocation of ^15^N varied across the year (MP × organ: χ^2^ = 190.0, df = 28, *P* < 0.001) and this seasonality pattern in allocation differed between the sites (MP × site × organ, χ^2^ = 64.6, df = 28, *P* < 0.001), with clearer differences between growing season and cold season in Vassijaure, while both sites showed clear variation within the cold season. Surprisingly, a larger part of the recovered ^15^N moved up in the continuum FR–CRs–shoots during colder parts of the year than during the warmer periods, with the exception of late spring–winter in the snow‐rich site Vassijaure.

As such, the proportion of plant‐recovered ^15^N allocated to shoots compared to roots was higher for MPs during the cold season (September–June, 17.2 ± 3.1% in shoots) than during the growing season (July–August, 7.3 ± 1.7%; t = −2.5, df = 176, *P* = 0.014), in Vassijaure only. In both sites, it was higher for MPs during the snow‐covered period (November–May, Abisko: 13.9 ± 2.6%; Vassijaure: 20.5 ± 3.6%) than during the snow‐free part of the cold season (September–October, Abisko: 2.1 ± 0.7%, *t* = 3.2, df = 176, *P* = 0.001; Vassijaure: 2.6 ± 0.9%, *t* = 3.9, df = 176, *P* < 0.001). Again, only in Vassijaure was it lower for MPs during the cold part (November–March, 13.6 ± 3.8%) than during the warm part of the snow‐covered period (April–May, 29.1 ± 6.2%; *t* = −3.7, df = 176, *P* < 0.001).

The proportion of plant‐recovered ^15^N allocated to FR compared to CR was lower for MPs during the cold season (September–June, 50.9 ± 3.3% (FR), 31.8 ± 2.5% (CR)) than during the growing season (July–August, 70.5 ± 3.7% (FR), 22.2 ± 3.1% (CR), *t* = −4.4, df = 176, *P* < 0.001) in Vassijaure only. In Abisko only, it was lower for MPs during the snow‐covered period (November–May, 58.5 ± 3.9% (FR), 27.6 ± 2.9% (CR)) than during the snow‐free part of the cold season (September–October, 81.7 ± 5.8% (FR), 16.2 ± 6.0% (CR); *t* = 4.8, df = 176, *P* < 0.001) and higher for MPs during the cold part of the snow‐covered period (November–March, 60.6 ± 5.1% (FR), 23.9 ± 2.9% (CR)) than during the warm part of the snow‐covered period (April–May, 54.9 ± 6.1% (FR), 33.8 ± 6.0% (CR); *t* = −2.1, df = 176, *P* = 0.039) (Fig. 4).

### Seasonality of ^15^N‐recovery in soil extractable nitrogen

Absolute ^15^N‐recovery in the TDN was < 1% throughout the year (Figs 3a, S5), varied between MPs (χ^2^ = 77.1, df = 14, *P* < 0.001) and was slightly higher in Abisko than Vassijaure (site: χ^2^ = 4.6, df = 1, *P* = 0.032) without interaction. Relative ^15^N‐recovery of TDN also varied across the year (MP: χ^2^ = 87.5, df = 14, *P* < 0.001), without differences between sites or interaction. Neither absolute nor relative ^15^N‐recovery in TDN differed significantly between seasons or periods tested by our contrasts.

### Seasonality of estimated plant total inorganic N‐uptake

**Fig. 5:**
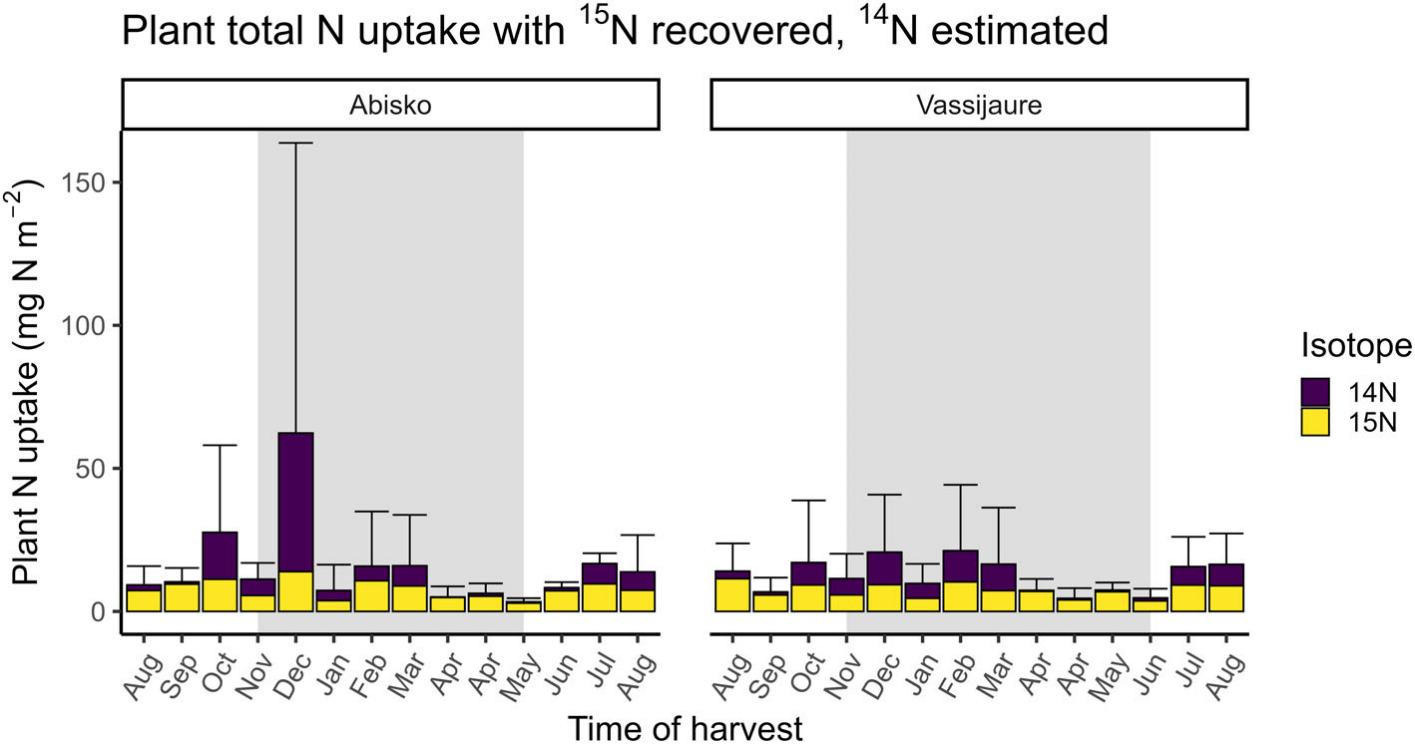
Year‐round seasonal patterns of estimated plant total N‐uptake in Abisko (left) and Vassijaure (right), 2019–2020. The total N‐uptake is the combined uptake of ^14^N + ^15^N, where the ^15^N is from measured recovery after ^15^N‐labelling and ^14^N is estimated based on ^15^N‐recovery and the average fractional abundance of ^15^N in the dissolved inorganic nitrogen pool (see Methods S1). Note that there is an early April and a late April measurement. The grey shading in the background indicates the snow‐covered period. Monthly bars refer to when the experimental plots were harvested and represent estimated uptake over the preceding 3‐wk period between labelling and harvest. Error bars represent 95% confidence intervals (*n* = 5).

Because the inorganic N‐pool was higher in several of the early cold season months than in spring‐winter and summer (Fig. S6), and the estimated inverse isotopic ratio (^15^R^−1^ = ^14^N : ^15^N) of the soil N‐pool was therefore similar or larger in part of the cold season than in summer (Fig. S7), the plants took up a similar or higher ratio of ^14^N per ^15^N recovered in part of the cold season than in summer. Despite lower plant biomass during the snow‐covered period (Fig. S4), differences across the year in estimated plant total inorganic N‐uptake m^−2^ (recovered ^15^N and estimated ^14^N) were even more pronounced (χ^2^ = 49.6, df = 13, *P* < 0.001) than for absolute ^15^N‐recovery (Fig. 5 vs Fig. S2), while there were no differences across sites, nor interaction. In Abisko, the estimated plant total inorganic N‐uptake during 3‐wk periods of each season did not differ between MPs in the growing season vs the cold season (average of 12.0 ± 1.5 vs 16.5 ± 4.3 mg N m^−2^), but it was 19% higher for MPs during the snow‐free than during the snow‐covered part of the cold season (19.0 ± 6.0 vs 15.9 ± 1.9 mg N m^−2^; *t* = −2.4, df = 52, *P* = 0.021), and 4.6 times higher for MPs during the cold part compared to the warm part of the snow‐covered period (22.5 ± 8.1 vs 4.9 ± 0.7 mg N m^−2^; *t* = 4.3, df = 52, *P* < 0.001). In Vassijaure, estimated plant total inorganic N‐uptake was 33% higher for MPs in the growing season vs the cold season (15.4 ± 2.0 vs 11.6 ± 1.5 mg N m^−2^; *t* = 2.3, df = 52, *P* = 0.027), did not differ between MPs during the snow‐free vs the snow‐covered part of the cold season (11.9 ± 0.02 vs 11.5 ± 1.7 mg N m^−2^), and was 2.7 times higher for MPs during the cold part compared to the warm part of the snow‐covered period (15.9 ± 2.8 vs 6.0 ± 0.6 mg N m^−2^; *t* = 3.2, df = 52, *P* = 0.003).

Arctic seasons are not equal in length: the growing season lasted for 9–12 wk in Vassijaure and Abisko, respectively, and the cold season 40–43 wk. Combined with the seasonal differences in estimated total plant N‐uptake ‘rates’ above, the cold season therefore contributed 3.6–4.6 times as much as the growing season to the estimated annual total plant N‐uptake (Abisko: 220.3 ± 67.0 vs 48.0 ± 5.7 mg N m^−2^; Vassijaure: 166.0 ± 23.1 vs 46.1 ± 6.2 mg N m^−2^). In other words, *c*. 78.3–82.1% of the estimated annual total plant N‐uptake occurred during the cold season (Vassijaure–Abisko), and as much as 49.6–56.0% during deep‐winter (November–March: Abisko: 150.2 ± 66.7 mg N m^−2^; Vassijaure: 106.1 ± 23.3 mg N m^−2^; Table S3).

## Discussion

Through consecutive injections of ^15^N‐tracer and harvests every month over a whole year, we showed that plants in low Arctic tundra display a clear capability for acquiring inorganic N year‐round, with – surprisingly – mostly similar recovery and estimated uptake rates in the cold season as in summer, and the highest cold‐season recovery and estimated uptake rates in deep‐winter. The majority of N taken up by plants was retained belowground, but a higher fraction was allocated to aboveground biomass during the snow‐covered period. Our results have important implications for our understanding of the role of the cold season in nutrient acquisition of plants in northern ecosystems and the seasonality of their functioning.

### Year‐round uptake of inorganic N by arctic plants

In line with part of our first hypothesis, plants recovered the ^15^N‐label throughout the year, which demonstrates that arctic plants have a capacity to take up inorganic N even in deep‐winter. Surprisingly, we found only a few indications of higher ^15^N‐recovery or estimated inorganic N‐uptake in the growing season than during the cold season. Instead, both absolute plant ^15^N‐recovery and estimated plant inorganic N‐uptake strongly varied within the cold season or the snow‐covered period, with often clearly higher recovery or estimated uptake in the cold deep‐winter than in the warmer spring‐winter. Monthly mean soil temperatures reached their minimum in March (−1.0°C, Abisko) and April (−0.86°C, Vassijaure), that is, in late deep‐winter or early spring‐winter, but then gradually increased again. It therefore seems unlikely that the still relatively mild soil temperatures alone have caused the lower ^15^N‐recovery or estimated N‐uptake throughout spring‐winter (Fig. S2). Instead, the plants may have been limited in inorganic N‐uptake during spring‐winter by the much lower soil inorganic N‐pool and turnover compared to deep‐winter (Figs S6, S7). Further, it might be possible that the plants' intrinsic capacity for N‐uptake was lower towards the end of the cold season, as N‐uptake is an active process and plant respiration at temperatures close to 0°C under snow can lead to a loss of stored non‐structural carbohydrates during the cold season (Saarinen & Lundell, 2010) while photosynthesis might not have been possible yet until the very end of the snow‐covered period. To a lesser extent, the lower (absolute) plant ^15^N‐recovery and estimated plant total inorganic N‐uptake in spring–winter than in deep‐winter may also have been caused by some of the label having leached out during snowmelt, as total ecosystem ^15^N‐recovery was also somewhat lower in spring‐winter than in deep‐winter (Fig. 3), which suggests that our measurements may have somewhat underestimated spring‐winter ^15^N‐recovery and estimated plant total inorganic N‐uptake.

Several studies have shown that plant N‐uptake in the Arctic is high during the period of snowmelt (Bilbrough *et al*., 2000; Grogan & Jonasson, 2003; Grogan *et al*., 2004; Miller *et al*., 2009; Edwards & Jefferies, 2010) and in late autumn (Larsen *et al*., 2012; Blume‐Werry *et al*., 2019), and that a single pre‐winter ^15^N‐labelling is quickly taken up by plants and microbes and then little re‐distributed until the next spring (Grogan & Jonasson, 2003; Larsen *et al*., 2012). However, continued nutrient uptake during the coldest and darkest part of the Arctic winter has not been shown before. Here, we applied ^15^N monthly throughout the year, each time followed by plant and soil harvests 3 wk later, demonstrating that short‐term plant ^15^N‐recovery was not lower during the cold season than during the growing season, as is usually assumed. Taking the different lengths of the seasons (Fig. 1, Table S3) into account, we estimate that more than 75% of the annual plant inorganic N‐uptake may take place during the 9–10‐month‐long cold season and approximately half during deep‐winter (November until March). This emphasises the importance of the cold season in meeting annual plant nitrogen requirements even in the Arctic (Grogan & Jonasson, 2003; Andresen & Michelsen, 2005; Larsen *et al*., 2012; Riley *et al*., 2021).

Plant inorganic N‐uptake depends on external limitations, such as N‐availability, but is also an active, energy‐consuming process, involving nitrate and ammonium transporters in the roots (Reid & Hayes, 2003; Kiba & Krapp, 2016), which may provide potential intrinsic problems during the cold, dark Arctic winter. Importantly, snow was deep enough and arrived early enough at both sites to stabilise soil temperatures near 0°C throughout the cold season – despite air temperatures as low as −26°C. As a consequence, the soils did not freeze solid at these low‐Arctic sites throughout most of the winter in the year of our measurements, and there was apparently enough liquid water available in soil pores to sustain a supply of similar or even higher background inorganic ^14^N‐concentrations, despite being low, as the injected ^15^N‐concentrations during a large part of the cold season (Fig. S7). Uptake of N might further be limited in the cold season by strong light limitations of photosynthesis by the absence of direct sunlight or thick snow cover, even if the photosynthetic capacity of overwintering evergreen plants may remain intact under the snow (Saarinen & Lundell, 2010). However, storage organs for energy and nutrients, such as in rhizomes, thick stems and large root systems, may give the – mostly perennial – arctic plants the capacity to take up N even when C‐fixation is limiting (Shaver *et al*., 1992; Blume‐Werry *et al*., 2019). Taken together, plant N‐acquisition may thus be temporally disconnected from photosynthesis and continue during a season as seemingly unfavourable as the long, cold, and dark Arctic winter. These external and internal conditions at our sites apparently sufficed for plants to actively take up inorganic N at similar or higher rates in most of the cold season as in summer (Fig. 5).

Our sampling patches were selected to include a variety of plant species from different growth forms representative of low‐Arctic tundra. Further, we applied dual‐labelled ^15^N‐NH_4_NO_3_ to ensure that a broad range of plant species could acquire the label. At least some species in our mixed tundra vegetation (Table S1) thus seem to be able to take up either form of inorganic N during the cold season. With our approach, we cannot, however, exclude that some of the ^15^N reached the plants after first having been taken up and transformed by microbes or via mycorrhizal fungi. Soil microbes can lose part of their acquired N during the winter due to turnover (Larsen *et al*., 2012). Mycorrhizal structures may be long‐lived (Clemmensen *et al*., 2006), but it is currently not known whether the plant–mycorrhizal exchange of C and N continues during the Arctic winter. Further, our estimations of the total plant *inorganic* N‐uptake represent only part of the total N‐uptake of arctic plants, as many species can take up *organic* N‐forms, either directly or through associations with mycorrhizal fungi (Iversen *et al*., 2015). It has been suggested that plant preferences for different N‐forms change seasonally, to match the most abundant N‐forms in the soil, with the highest organic (glycine) uptake in an alpine meadow in autumn compared to spring and summer (Broadbent *et al*., 2024). An important next step would be to quantify whether such high uptake of organic N also persists after the abundant autumn litterfall and through the snow‐covered season, or if high deep‐winter plant uptake in cold ecosystems is exclusive to inorganic N‐forms.

Spatial and temporal variation in nutrient availability and differences in the ability of different plant species to exploit these resources influence competition and vegetation composition (McKane *et al*., 2002; Keuper *et al*., 2017). Uptake of nutrients in excess of immediate growth requirements during times of relatively high availability, to support growth when availability is lower, has been called ‘luxury consumption’ (Chapin, 1980; van Wijk *et al*., 2003). It has been suggested that such luxury consumption of N by the existing, typically slow‐growing tundra vegetation can prevent invasion by faster‐growing species without such capacities, through depletion of the available N (van Wijk *et al*., 2003). While we do not know whether the ability for winter N‐uptake differs between the species in the mixed tundra studied here, our results suggest that temporal niche differentiation and its consequences for vegetation stability through luxury consumption should be considered in a year‐round perspective. Maximising off‐season N‐uptake might not only be crucial for the generally N‐limited tundra plants to maintain a high growth and C‐sequestration during summer, but also for the stability of arctic tundra vegetation.

### Higher aboveground N‐allocation under snow

As expected, the majority of plant‐recovered ^15^N was retained in the root biomass throughout the year (58.5–99.0%)—partly an effect of the greater belowground vs aboveground biomass (Fig. S4). In contrast to our second hypothesis, however, the relative allocation to aboveground tissues was twice as high during the cold season as in summer in Vassijaure, and more than six times as high during the snow‐covered as the snow‐free parts of the cold season in Abisko. The allocation to FR was, meanwhile, much lower during these periods. FR biomass was much lower in the cold season than in summer (Fig. S4), suggesting overwinter FR mortality. The reduced retention of the acquired N in FR during the coldest part of the winter might therefore be caused by the lower root biomass or be an active strategy to avoid losing the acquired N through the FR mortality. Growth of FR is crucial to sustain nutrient acquisition in the generally N‐limited tundra (Iversen *et al*., 2015) and occurs from early spring through late autumn, but then mostly ceases during the winter (Blume‐Werry *et al*., 2016, 2019). Our results suggest that the growing FR are such a strong N‐sink during the summer that very little of the acquired N is transferred further into the CR and shoot compared to the cold season, and that the N that is used for leaf production and shoot growth primarily comes from storage.

In autumn‐winter and deep‐winter, more and more of the acquired N is available for further transfer and storage (Bret‐Harte *et al*., 2002). A larger proportion of recovered ^15^N was then found in the CR, while the aboveground allocation was initially small but increased through the snow‐covered period (Fig. 4). Our tundra heath patches contained a mixture of growth forms, but were overall dominated by evergreen and deciduous shrubs. While all functional groups, including graminoids and forbs, acquired ^15^N during the cold season (data not shown), evergreen shrubs allocate relatively much of their annual production to leaves and deciduous shrubs to stems, whereas graminoids allocate more of their biomass to roots and rhizomes (Hobbie, 1996). The location of the main storage tissues in evergreen and deciduous shrubs might therefore dominate the observed higher aboveground ^15^N‐allocation during the snow‐covered period.

### Continued microbial inorganic N‐acquisition through deep‐winter

It is well known that microbial growth can continue at subzero temperatures down to −17°C (Mikan *et al*., 2002; Panikov & Sizova, 2007) and respiration down to −39°C (Panikov *et al*., 2006), especially when unfrozen water is not limiting (Öquist *et al*., 2009). Microbial respiration and breakdown of organic matter can therefore remain relatively high in the Arctic through the cold season (Hobbie & Chapin, 1996; Zona *et al*., 2016; Natali *et al*., 2019). We add to this that microbial ^15^N‐recovery can also remain active throughout the cold season and, more surprisingly, that it can even peak during deep‐winter at sites like ours, when temperatures are relatively mild under the snow throughout the cold season. Also consistent with other studies in cold ecosystems (Jonasson *et al*., 1999; Schmidt *et al*., 2002; Clemmensen *et al*., 2008; Larsen *et al*., 2012), the soil microbes accounted for 72.4–94.1% of the ecosystem‐recovered ^15^N throughout the year (Fig. 3b). Interestingly, however, this relative distribution between microbes and plants did not vary throughout the year. This suggests that the competitive strengths of these two groups for inorganic N did not vary much throughout the year either.

### The role of snow depth and duration in the seasonality of inorganic N‐uptake

Snow is an important modifier of the microenvironment experienced by small‐statured tundra plants and soil microbes, through its strong insulating and reflective properties (Williams & Smith, 1989). Maximum snow depth was almost twice as deep and snow‐cover duration a month longer in Vassijaure than in Abisko, but we found no significant interactions between site and time of year for ^15^N‐recovery and estimated plant total inorganic N‐uptake, except for allocation patterns. In contrast to our third hypothesis, the sites did not differ greatly in winter soil temperatures. Snow depths between 20 and 50 cm were deep enough to largely decouple air and soil temperatures through the entire snow‐covered period at these low‐Arctic sites (Fig. 2) and keep soil temperatures in a range where microbes (Brooks *et al*., 1996; Schimel *et al*., 2004; McMahon *et al*., 2009) – but clearly also plants – can be active and take up inorganic N even during deep‐winter. Interestingly, only in our high‐snow site, Vassijaure, did we find any significant differences in plant N‐dynamics between growing season and cold season: ^15^N‐allocation to shoots was lower, to FR higher, and estimated plant total inorganic N‐uptake (per m^2^) was also higher for MPs during the growing season than during the cold season. The 33% higher estimated total plant N‐uptake in the growing‐season MPs was likely caused by seasonal differences in biomass (Fig. S4) and not by higher ^15^N‐recovery (Fig. S2), as estimated plant total inorganic N‐uptake was not different between MPs in the summer and the cold season if expressed per plant dry weight (DW) or FR DW instead of per m^2^ (Fig. S8). Tundra plants in late‐snowmelt areas are adapted to speed up their growth and produce their biomass despite the shorter summer (Wipf, 2010), which, combined with similar N‐uptake rates per DW, may thus enable equal total growing‐season N‐uptake as in early snowmelt areas. Compression of the growing season length by later snowmelt may thus not necessarily reduce the total growing season N‐uptake to the same extent (Table S3).

The degree to which the winter N‐uptake by plants and microbes observed at our relatively mild low‐Arctic tundra sites is realised in other parts of the Arctic or under future climate conditions will likely vary. The tundra biome covers 11% of the Northern Hemisphere land area (Virtanen *et al*., 2016), with large‐scale spatial gradients in duration and harshness of the cold season (Ernakovich *et al*., 2014) and small‐scale variation in terrain and vegetation, causing variation in temperatures and snow cover thickness and duration (Sturm *et al*., 2005; Graae *et al*., 2012; Kropp *et al*., 2021). For locations in the Arctic with much lower winter air temperatures, snow depth and onset are crucial for determining soil temperature and liquid water availability (Coulson *et al*., 1995; Zhang, 2005; Elberling, 2007) and might not be sufficient to achieve continued N‐uptake through the winter. On the other hand, given the high plant and microbial ^15^N‐recovery in deep‐winter observed at both our sites, it seems likely that other sites with strong decoupling between air and soil temperature, such as habitats with similar snow cover in other low Arctic areas or deeper, early snow cover in the high Arctic, will also show at least some winter N‐uptake. The next step would be to quantify the N‐uptake potential of plants across Arctic sites that differ more strongly in winter temperatures and snow cover, and thus winter soil conditions.

### Conclusion

The Arctic winter is cold and dark and extends through a large part of the year (Olsson *et al*., 2003). Here, we demonstrate that tundra plants' inorganic N acquisition continues year‐round and can even peak in deep‐winter under sufficient snow cover. Our results, therefore, challenge the general assumption that arctic plants remain dormant during the winter. Much of the ecological research in the Arctic focusses on the ‘growing season’, which is generally perceived as the part of the year when visible growth or aboveground plant processes such as biomass production take place (Körner *et al*., 2023), and is very short in most of the Arctic. Instead, our results contribute to the recognition that the duration of the ‘active season’ in the Arctic may not only vary strongly between aboveground and belowground growth (Blume‐Werry *et al*., 2016) but may be much longer than the aboveground growing season for other vital plant processes too. Studies on plant functioning and their responses to climate change should therefore include a whole‐year perspective, even in the Arctic. Understanding nutrient acquisition of plants and microbes in a year‐round perspective and their responses to the rapid changes in winter temperatures, snow cover thickness, and duration (Thompson & Wallace, 2001; IPCC, 2021) will ultimately be critical for predicting future plant productivity and C and N cycling in the Arctic.

## Competing interests

None declared.

## Author contributions

ED conceived the idea. ED, NL and JW designed and set up the study. EASA, JW, NL, ED and FG planned and conducted the field and lab work. AM performed analysis of soil‐extracted N‐isotopes. EASA, ED, JO and CF performed the data analysis. EASA, ED and GBW made the tables and figures. EASA drafted the manuscript to which all authors contributed with writing, validation of text and analysis, and approval of the final version.

## Disclaimer

The New Phytologist Foundation remains neutral with regard to jurisdictional claims in maps and in any institutional affiliations.

## Supporting information


**Fig. S1** Site locations and example patch pictures.
**Fig. S2** Seasonal plant ^15^N absolute recovery.
**Fig. S3** Seasonal microbial ^15^N absolute recovery.
**Fig. S4** Seasonal plant biomass aboveground and belowground.
**Fig. S5** Seasonal total dissolved nitrogen ^15^N absolute recovery.
**Fig. S6** Seasonal soil inorganic N (ammonium and nitrate).
**Fig. S7** Estimated seasonal inverse fractional abundance (*F* ^−1^) and isotopic ratio (*R* ^−1^).
**Fig. S8** Estimated plant total N‐uptake per g dry weight (DW) sample and per g DW fine roots.
**Methods S1** Calculation of recovery of ^15^N and estimation of plant total inorganic N‐uptake.
**Table S1** Vegetation composition of the sites.
**Table S2** Estimated inverse isotopic ratio (*R* ^−1^) and fractional abundance (*F* ^−1^) of ^15^N in the soil inorganic N‐pool.
**Table S3** Seasonal distribution of estimated annual total plant inorganic N‐uptake.
Please note: Wiley is not responsible for the content or functionality of any Supporting Information supplied by the authors. Any queries (other than missing material) should be directed to the *New Phytologist* Central Office.

## Data Availability

The data that support the findings of this study are openly available at Zenodo, doi: 10.5281/zenodo.18328841. Code for analysis can be found at https://github.com/the‐esherman/Project_I_15N_seasonality.
