# Supplementary material for: Arctic plants can take up inorganic nitrogen year‐round
Source: New Phytol. 2026 May 18;251(4):1694–708. doi: 10.1111/nph.71273 (PMC13373808; doi:10.1111/nph.71273)
Supplement: Supplementary file 1 — Fig. S1 Site locations and example patch pictures. Fig. S2 Seasonal plant 15N absolute recovery. Fig. S3 Seasonal microbial 15N absolute recovery. Fig. S4 Seasonal plant biomass aboveground and belowground. Fig. S5 Seasonal total dissolved nitrogen 15N absolute recovery. Fig. S6 Seasonal soil inorganic N (ammonium and nitrate). Fig. S7 Estimated seasonal inverse fractional abundance (F −1) and isotopic ratio (R −1). Fig. S8 Estimated plant total N‐uptake per g dry weight (DW) sample and per g DW fine roots. Methods S1 Calculation of recovery of 15N and estimation of plant total inorganic N‐uptake. Table S1 Vegetation composition of the sites. Table S2 Estimated inverse isotopic ratio (R −1) and fractional abundance (F −1) of 15N in the soil inorganic N‐pool. Table S3 Seasonal distribution of estimated annual total plant inorganic N‐uptake. Please note: Wiley is not responsible for the content or functionality of any Supporting Information supplied by the authors. Any queries (other than missing material) should be directed to the New Phytologist Central Office. [file NPH-251-1694-s001.pdf]

Supplementary material for

Arctic plants can take up inorganic nitrogen year-round

Emil Alexander Sherman Andersen[1], Gesche Blume-Werry[2], Chenxin Feng (冯晨辛)[1,3], Friederike Gehrmann[1], Niki Leblans[1], Anders Michelsen[4], Johan Olofsson[2], Josefine Walz[1], Ellen Dorrepaal[1]

[1] *Climate Impacts Research Centre, Department of Ecology, Environment and Geoscience , Umeå University, 981 07 Abisko, Sweden*
[2] *Department of Ecology, Environment and Geoscience, Umeå University, 901 87 Umeå, Sweden*
[3] *Faculty of Environmental and Agricultural Sciences, Agricultural University of Iceland, Hvanneyri, IS-311 Borgarness, Iceland*
[4] *Terrestrial Ecology Section, Department of Biology, University of Copenhagen, 2100 Copenhagen Ø, Denmark*

Corresponding author: Emil A.S. Andersen
Email: ea.sherman@proton.me
https://orcid.org/0000-0003-3745-5044

Article acceptance date: 1 May 2026

The following Supplementary material is available for this article:

Supplementary methods: Calculation of recovery of ^{15}N and estimation of plant total inorganic N-uptake...........p. 2–5

Suppl. Tables S1–S3

Table S1: Vegetation composition of the sites...........p. 6

Table S2: Estimated inverse isotopic ratio (R^{-1}) and fractional abundance (F^{-1}) of ^{15}N in the soil inorganic N-pool...........p. 7

Table S3: Seasonal distribution of estimated annual total plant inorganic N-uptake…...p. 8

Suppl. Figures S1–S8:

Fig. S1: Site locations and example patch pictures...........p. 9

Fig. S2: Seasonal plant ^{15}N absolute recovery...........p. 10

Fig. S3: Seasonal microbial ^{15}N absolute recovery...........p. 11

Fig. S4: Seasonal plant biomass above- and belowground...........p. 12

Fig. S5: Seasonal total dissolved nitrogen ^{15}N absolute recovery...........p. 13

Fig. S6: Seasonal soil inorganic N (ammonium and nitrate)...........p. 14

Fig. S7: Estimated seasonal inverse fractional abundance (F^{-1}) and isotopic ratio (R^{-1}) p. 15

Fig. S8: Estimated plant total N-uptake per g dry weight (DW) sample and per g DW fine roots...........p.16

References...........p. 17

Supplementary methods: Calculation of recovery of ^{15}N and estimation of plant total inorganic N-uptake

Atom%^{15}N of labelled and non-labelled fractions was calculated from the δ^{15}N values with the equation (s1):

$$AP^{15}N = \frac{100}{1 + \frac{1/^{15}N_{ref}}{1 + \delta^{15}N/1000}} \tag{s1}$$

Where $^{15}N_{ref}$ is the ^{15}N/^{14}N fractional abundance in isotopic reference equal to 1/272.
For all plant organs (shoots, coarse roots, and fine roots) and total dissolved nitrogen (TDN) recovery of ^{15}N-label (%R, % of added ^{15}N) was calculated using (s2):

$$\%R = \frac{\frac{(AP^{15}N_{lab} - AP^{15}N_{NatAb})}{100} \times \frac{\omega_N\%}{100} \times mass}{^{15}N_{inj}} \times 100 \tag{s2}$$

Where $AP^{15}N_{lab}$ and $AP^{15}N_{NatAb}$ are labelled and unlabelled atom%^{15}N (AP = atom%) respectively, ω_N is the N concentration in per cent of dry weight (DW), *mass* is the plant biomass, or soil mass for TDN, of the analysed fraction (in g DW per core), and $^{15}N_{inj}$ is the injected ^{15}N in g per core. Note that because of small biomass and often unequal distribution or absence of many non-dominant species across the 170 patches of vegetation, as well as the small portion of the (fine) roots that could be identified at species or growth-form level (from roots remaining attached to the shoot of individual plants during harvest), we did not analyse the plant biomass and isotopic recovery data by species or growth form, but only pooled by whole plant community.

Recovery of the ^{15}N-label in the soil microbial N-pool was calculated using a slightly modified equation (s2), taking K_{EN} into account.

$$\%R = \frac{\{^{15}N_{lab}\} - \{^{15}N_{NatAb}\}}{^{15}N_{inj}} \times 100 \tag{s3}$$

Where

$$\{^{15}N_z\} = \frac{(AP^{15}N_{SEF_z} \times \omega_{NSEF_z} - AP^{15}N_{SE_z} \times \omega_{NSE_z}) \times mass}{K_{EN}} \tag{s4}$$

SE and *SEF* are the non-fumigated and fumigated extracts, respectively, while *z* represents labelled (*lab*) and natural abundance (*NatAb*) in the same generalised formula. *Mass* is the soil mass DW of the whole core, as estimated as if root-sorted:

$$mass = \frac{soil_{subRF}}{soil_{sub}} \times soil_{total} \times DW_{frac} \quad (s5)$$

Where $soil_{sub}$ is the unsorted subsampled soil weight, $soil_{subRF}$ is the root-sorted subsample weight, $soil_{total}$ is the total soil core weight, and DW_{frac} is the dry weight fraction calculated as DW to fresh weight (FW) from gravimetric soil moisture samples.

To estimate plant total inorganic N-uptake based on ^{15}N-recovery, we estimated the dissolved inorganic N-pool during the 3-week labelling period and the inverse ^{15}N isotopic ratio ($^{15}R^{-1} = {}^{14}N/{}^{15}N$) of this pool. The average ^{15}R was estimated based on two linear interpolations: First to estimate the dissolved inorganic N concentration at injection, then to estimate the average isotopic ratio during the three weeks between injection and subsequent harvest.

Because of practical constraints, we did not take samples at the injection point to determine the inorganic N concentration and isotopic signature. Instead, we estimated the inorganic N concentration at injection by linear interpolation between the harvest one week prior to and the next harvest three weeks after the injection, accounting for the one week between the previous harvest and the subsequent labelling. The injected N was added to the interpolated inorganic N concentration at injection to get the total dissolved inorganic N concentration at injection (see figure below, note that sometimes, particularly in spring-winter and late summer, the injected ^{15}N constituted up to 100% of the N-pool at labelling).

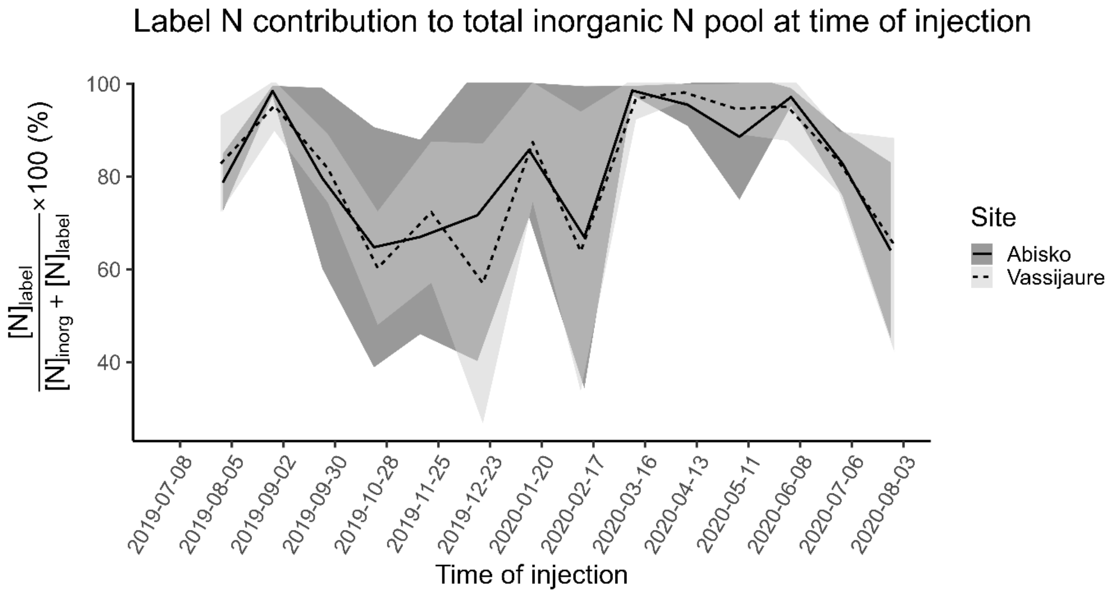

Injected ^{15}N as a fraction of the total inorganic N-pool (background inorganic N + injected N) at the time of injection. The inorganic N-pool at injection is interpolated from the inorganic N-pools at the two harvests, one week before and three weeks after injection. Error shading represents 95% confidence intervals (n = 5).

Secondly, we estimated inorganic ^{15}N and ^{14}N concentrations at injection and at harvest to be able to calculate the average isotopic ratio during the three weeks in between. At injection, the ^{15}N enrichment (atom%, AP) of the inorganic N-pool is a combination of the natural abundance of inorganic N (AP_{NatAb}) and the injected label ^{15}N (AP_{label}), which were both known. The natural abundance was based on the two control harvests in September 2019 and March 2020 (see Methods). Using the mass balance equation (Hayes, 2004),

$$m_\Sigma F_\Sigma = m_1 F_1 + m_2 F_2 \tag{s6}$$

where m is the molar quantity or mass, and F is the fractional abundance ($F \times 100 = AP$), we calculated the enrichment of the soil inorganic N at injection. Using the N concentration ([N]) for m and AP for F, we arrive at the following (s7)

$$AP_{inorg0\_\&\_label} \times [N]_{inorg0\_\&\_label} = AP_{NatAb} \times [N]_{soil\_inorg0} + AP_{label} \times [N]_{label} \tag{s7}$$

Where $AP_{inorg0\_\&\_label}$ is the combined AP of the inorganic pool before labelling, and the label $[N]_{inorg0\_\&\_label}$ is the concentration of inorganic N from both soil and label, $[N]_{soil\_inorg0}$ is the soil inorganic N concentration before labelling, $[N]_{label}$ is the label N concentration. All concentrations are in µg N per g DW.

By rearranging equation (s7), we arrive at equation (s8):

$$AP_{inorg0\_\&\_label} = \frac{AP_{NatAb} \times [N]_{soil\_inorg0} + AP_{label} \times [N]_{label}}{[N]_{inorg0\_\&\_label}} \tag{s8}$$

Because of analytical constraints and the large number of samples in this study, the enrichment of the inorganic N-pool at harvest (AP_{inorgH}) was not measured directly. Instead, we therefore performed a sensitivity analysis and analysed how two assumed distributions of the remaining ^{15}N-label over the different parts of the extractable N-pool at harvest would affect our total N-uptake estimations. As such, we estimated a range based on the measured enrichment of TDN (AP_{TDN}) and two different scenarios of how the remaining label would have been distributed between inorganic and organic N. The lower limit of enrichment of the inorganic N-pool would be equal to the natural abundance (AP_{NatAb}), assuming that AP_{inorgH} would not be more depleted than natural abundance. That is, all the label remaining in the TDN would have ended up in the organic N (AP_{org}). For the upper limit, we assumed that the

organic N-pool was similar to natural abundance ($AP_{org} = AP_{NatAb}$), and derived the upper limit of enrichment of the inorganic N-pool by using and re-arranging (s7):

$$AP_{inorgH} = \frac{AP_{TDN} \times [N]_{TDN} - AP_{NatAbu} \times [N]_{org}}{[N]_{inorgH}} \quad \text{(s9)}$$

This upper limit has a cap, in that we assume the AP_{inorgH} does not exceed AP_{label} (98.7%).

From the injection point $AP_{inorg0\_\&\_label}$ and harvest AP_{inorgH}, we calculated the concentration of inorganic ^{15}N and ^{14}N at both time points (injection and harvest), and then calculated the average across both time points for the concentrations of inorganic [^{15}N] and [N] (= [^{15}N] + [^{14}N]). From these, we could calculate the average fractional abundance (^{15}F) of the inorganic N-pool, using the basic equation (s10):

$$^{15}F = \frac{[^{15}N]}{[N]} \quad \text{(s10)}$$

^{15}R was then calculated using the equation from Hayes (2004):

$$^{15}R = \frac{^{15}F}{(1 - {^{15}F})} = \frac{^{15}N}{^{14}N} \quad \text{(s11)}$$

Inverting (s11), we get R^{-1} and we have an estimate of how much inorganic ^{14}N followed along with each ^{15}N recovered. This is a conservative estimate, assuming no preferential uptake of the lighter isotope ^{14}N over ^{15}N. Since the higher (upper) estimate and the low estimate in our sensitivity analysis are very similar (Table S2), we opted to continue using the high estimate—that is, high enrichment in the inorganic N-pool on average over the labelling period—for calculating plant total N-uptake.

Finally, total plant inorganic N-uptake was calculated based on ^{15}N recovered in plants (excess atom%, APE) and the high estimate F^{-1}: APE × F^{-1}, while plant total ^{14}N-uptake was calculated by APE and the high estimate R^{-1}: APE × R^{-1}. The final estimated plant total N-uptake was then divided by the total plant biomass of the core and multiplied by the plant biomass per m^2 to get to the results presented in Fig. 5.

Supplementary Tables

Suppl. Table S1: Vegetation composition of the patches of low-Arctic tundra used for ^{15}N-labelling. Averages of percentage cover estimates and their summed cover per functional group of 75 patches (10 × 10 cm^2) in Abisko and 70 in Vassijaure in July 2019.

**Species or Growth Form**	**Cover in Abisko** (% of ground area)	**Cover in Vassijaure** (% of ground area)
**Evergreen shrubs**	**41.2**	**40.6**
Andromeda polifolia L.	8.1	6.4
Cassiope tetragona (L.) D.Don	0	0.1
Dryas octopetala L.	0	0.5
Empetrum hermaphroditum Hagerup	31.6	28.8
Kalmia procumbens (L.) Gift & Kron	0	2.1
Rhododendron lapponicum (L.) Wahlenb.	1.5	0
Vaccinium vitis-idaea L.	0	2.7
**Deciduous shrubs**	**17.9**	**14.1**
Arctostaphylos alpina (L.) Spreng.	1.9	0.4
Betula nana L.	0.2	0.3
Salix herbacea L.	0.3	0.1
Salix myrsinites L.	0.1	0
Vaccinium myrtillus L.	0	0.4
Vaccinium uliginosum L.	15.4	12.9
**Graminoids**	**18.4**	**13.3**
Calamagrostis lapponica Hook.	0	0.1
Carex nigra (L.) Reichard	12.3	13.1
Carex spp.	6.1	0.1
**Forbs**	**3.4**	**0.7**
Astragalus alpinus L.	0	0
Bartsia alpina L.	0.2	0
Bistorta vivipara (L.) Delarbre	0.3	0.5
Pedicularis lapponica L.	0	0.1
Pinguicula vulgaris L.	0.4	0
Rubus chamaemorus L.	0.3	0
Saxifraga aizoides L.	0.1	0
Tofieldia pusilla (Michx.) Pers.	2.1	0
**Other**		
Equisetum spp.	1.6	0
Bryophytes	62.9	71.5
Lichens	21.2	26.5

Suppl. Table S2: Estimated inverse isotopic ratio (R^{-1}) and fractional abundance (F^{-1}) of ^{15}N in the soil inorganic N-pool during uptake, as an average of the three-week labelling period. The high and low estimates refer to estimated enrichment (^{15}N) of the soil inorganic N pool. For details on how the low and high estimates were calculated, we refer to the Suppl. Methods. Months refer to when the experimental plots were harvested and represent the preceding three-week period between labelling and harvest. Values are given with 95% confidence intervals (CI).

Site	Measuring period	$[^{14}N]$ per $[^{15}N]$ as average over the labelling period: $R^{-1}=\frac{^{14}N}{^{15}N}$		[N] ($^{14}N + {}^{15}N$) per $[^{15}N]$ averaged over the labelling period: $F^{-1}=\frac{(^{14}N+{}^{15}N)}{^{15}N}$	
		High estimate Mean ± 95% CI	Low estimate Mean ± 95% CI	High estimate Mean ± 95% CI	Low estimate Mean ± 95% CI
Abisko	August (2019)	0.29 ± 0.10	0.29 ± 0.10	1.29 ± 0.1	1.29 ± 0.1
	September	0.08 ± 0.06	0.09 ± 0.06	1.08 ± 0.06	1.09 ± 0.06
	October	1.28 ± 1.52	1.28 ± 1.52	2.28 ± 1.52	2.28 ± 1.52
	November	1.11 ± 0.90	1.11 ± 0.90	2.11 ± 0.9	2.11 ± 0.9
	December	2.01 ± 2.91	2.04 ± 2.94	3.01 ± 2.91	3.04 ± 2.94
	January	0.67 ± 0.90	0.67 ± 0.90	1.67 ± 0.9	1.67 ± 0.9
	February	0.81 ± 1.03	0.81 ± 1.03	1.81 ± 1.03	1.81 ± 1.03
	March	0.70 ± 0.73	0.71 ± 0.73	1.7 ± 0.73	1.71 ± 0.73
	April (early)	0.03 ± 0.01	0.03 ± 0.01	1.03 ± 0.01	1.03 ± 0.01
	April (late)	0.24 ± 0.24	0.24 ± 0.24	1.24 ± 0.24	1.24 ± 0.24
	May	0.16 ± 0.21	0.16 ± 0.21	1.16 ± 0.21	1.16 ± 0.21
	June	0.15 ± 0.10	0.16 ± 0.10	1.15 ± 0.1	1.16 ± 0.1
	July	0.79 ± 0.38	0.80 ± 0.38	1.79 ± 0.38	1.8 ± 0.38
	August (2020)	0.74 ± 0.65	0.74 ± 0.66	1.74 ± 0.65	1.74 ± 0.66
Vassijaure	August (2019)	0.24 ± 0.17	0.24 ± 0.17	1.24 ± 0.17	1.24 ± 0.17
	September	0.19 ± 0.16	0.19 ± 0.16	1.19 ± 0.16	1.19 ± 0.16
	October	0.82 ± 0.41	0.82 ± 0.42	1.82 ± 0.41	1.82 ± 0.42
	November	1.11 ± 0.60	1.11 ± 0.60	2.11 ± 0.6	2.11 ± 0.6
	December	1.29 ± 1.22	1.30 ± 1.22	2.29 ± 1.22	2.3 ± 1.22
	January	1.06 ± 1.05	1.07 ± 1.05	2.06 ± 1.05	2.07 ± 1.05
	February	0.79 ± 0.90	0.79 ± 0.91	1.79 ± 0.9	1.79 ± 0.91
	March	0.89 ± 1.19	0.90 ± 1.20	1.89 ± 1.19	1.9 ± 1.2
	April (early)	0.05 ± 0.05	0.05 ± 0.05	1.05 ± 0.05	1.05 ± 0.05
	April (late)	0.12 ± 0.11	0.12 ± 0.11	1.12 ± 0.11	1.12 ± 0.11
	May	0.07 ± 0.06	0.07 ± 0.06	1.07 ± 0.06	1.07 ± 0.06
	June	0.27 ± 0.43	0.27 ± 0.43	1.27 ± 0.43	1.27 ± 0.43
	July	0.65 ± 0.33	0.65 ± 0.33	1.65 ± 0.33	1.65 ± 0.33
	August (2020)	0.76 ± 0.82	0.77 ± 0.82	1.76 ± 0.82	1.77 ± 0.82

Suppl. Table S3: Seasonal distribution of estimated annual total plant inorganic N-uptake in two low-Arctic tundra heaths in Abisko and Vassijaure between 1 August 2019 and 31 July 2020, and length of each season. Total inorganic N-uptake per season (mean ± SE) is based on summation of estimated total ^{15}N + ^{14}N-uptake per three-week measuring periods and of interpolated estimates for the week between each harvest and the next labelling (n = 5). Note that the N-uptake values for the three periods in the cold season do not exactly add up to the total cold-season value in Vassijaure due to small rounding differences during the plot-level calculations. Percentage uptake for each season is calculated relative to the estimated total annual uptake. See Fig. 1 for the approx. corresponding months of the different seasons in the Arctic year.

Site	Seasons and sub-seasons		Seasonal total plant N-uptake (mg N m^{-2})	Seasonal contribution to annual total plant N-uptake (%)	Season length (weeks)	Season length relative to total year (%)
Abisko	Cold season		220.3 ± 67.0	82.2	40	76.9
		Autumn-winter	50.6 ± 16.4	18.8	8	15.4
		Deep-winter	150.2 ± 66.7	56.0	20	38.5
		Spring-winter	19.6 ± 3.5	7.3	12	23.1
	Growing season / Summer		48.0 ± 5.7	17.9	12	23.1
Vassijaure	Cold season		166.0 ± 23.1	78.3	43	82.7
		Autumn-winter	31.8 ± 11.6	14.9	8	15.4
		Deep-winter	106.1 ± 23.3	49.6	20	38.5
		Spring-winter	30.0 ± 2.5	14.0	15	28.8
	Growing season / Summer		46.1 ± 6.2	21.5	9	17.3

Supplementary Figures

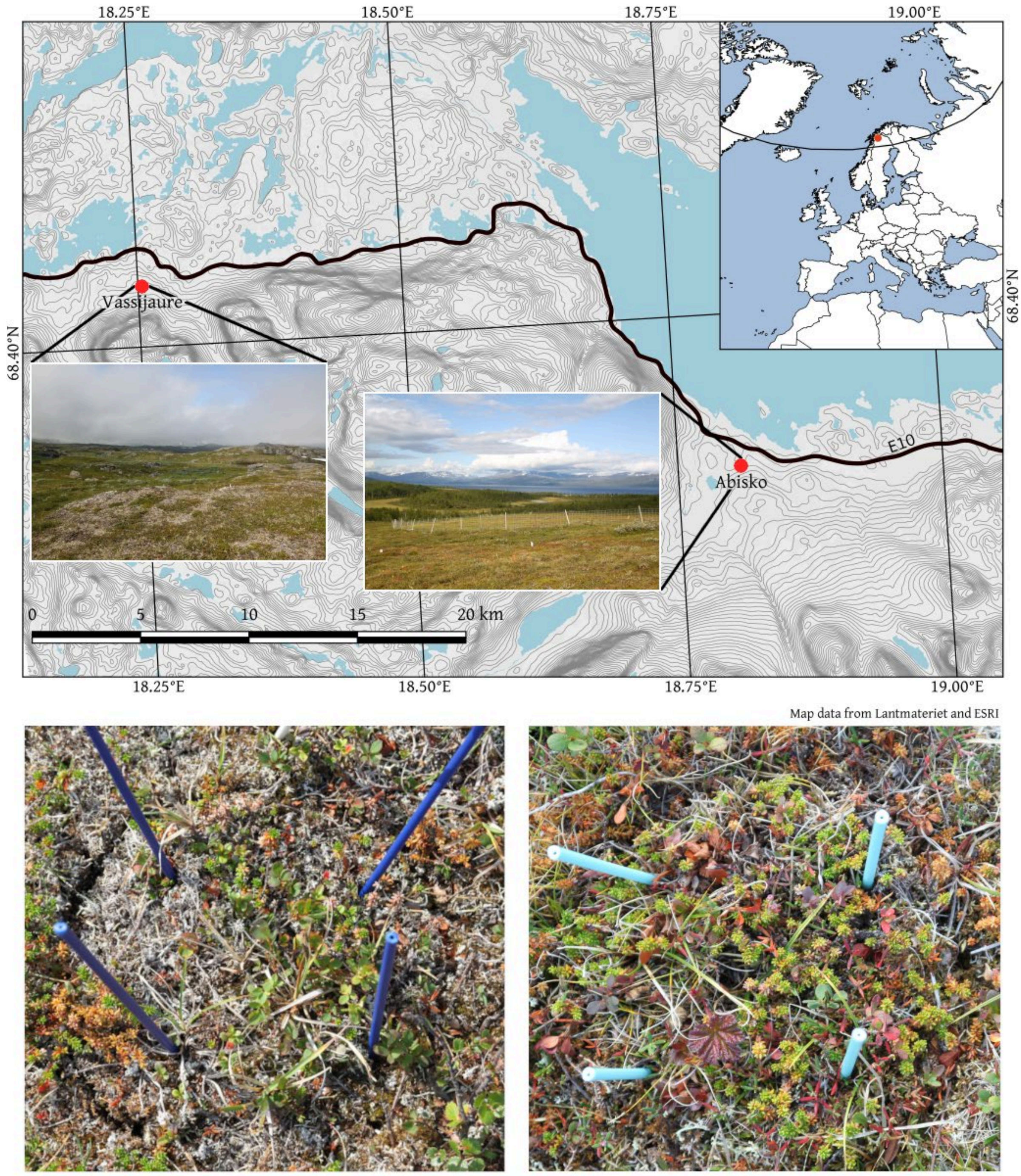

Supplementary Fig. S1: Location of site plots and examples of patches within plots. Upper panel: Site locations of Abisko and Vassijaure in Northern Sweden, approx. 200 km north of the polar circle. Lower panel: Examples of vegetation patches: Left: Vassijaure plot 5 patch 7 (designated to be used in measuring period 7, January 2020). Right: Abisko plot 4, patch 13 (designated to be used in measuring period 13, June 2020). Pictures were taken shortly after plot establishment in July and August 2019. Patches varied, but similar covers were found on both sites, i.e. vegetation similar to both pictures could be found at either site (see also Table S1 for vegetation cover).

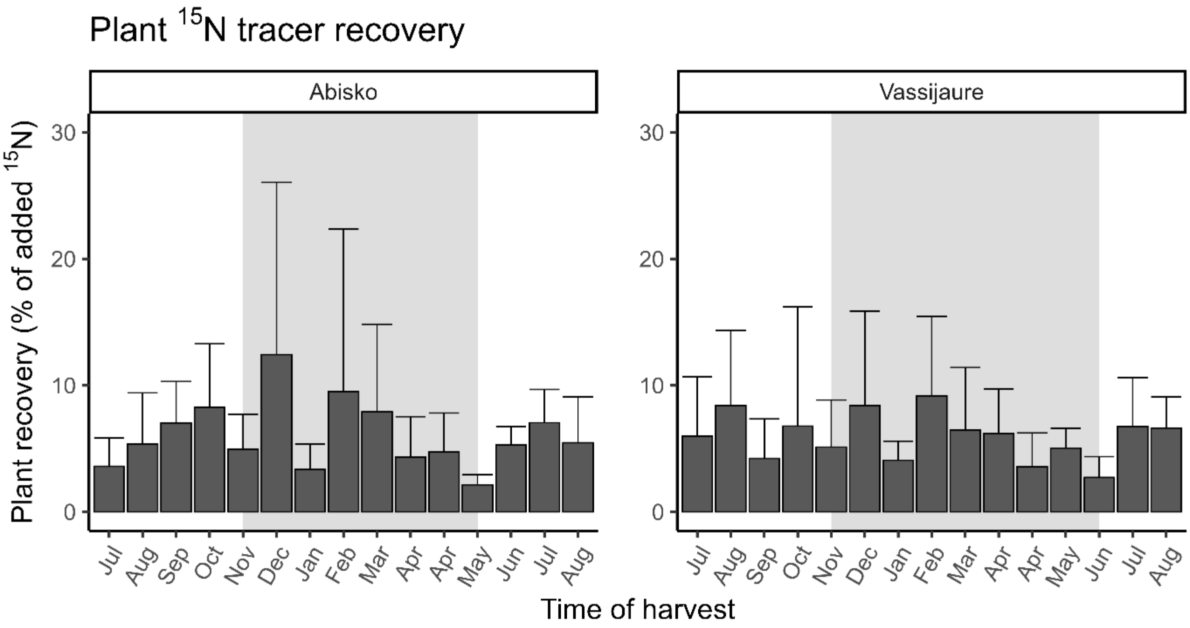

Supplementary Fig. S2: Year-round seasonal patterns of plant (absolute) recovery of ^{15}N tracer (% of addition) as whole plant community, including above- and belowground parts, in Abisko (left) and Vassijaure (right). Note that there is an early and a late April. The grey shading in the background indicates the snow-covered period. Monthly bars refer to when the experimental plots were harvested and represent estimated uptake over the preceding three-week period between labelling and harvest. Error bars represent 95% confidence intervals (n = 5).

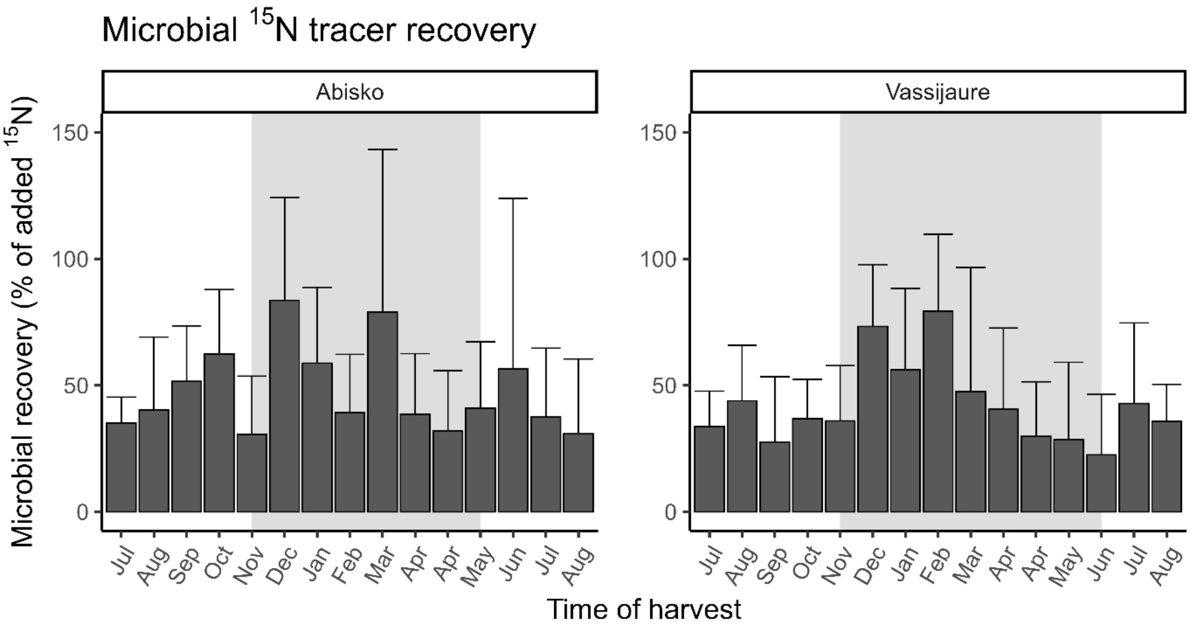

Supplementary Fig. S3: Year-round seasonal patterns of microbial (absolute) recovery of ^{15}N tracer (% of addition), in Abisko (left) and Vassijaure (right). Note that there is an early and a late April. The grey shading in the background indicates the snow-covered period. Monthly bars refer to when the experimental plots were harvested and represent estimated uptake over the preceding three-week period between labelling and harvest. Error bars represent 95% confidence intervals (n = 5).

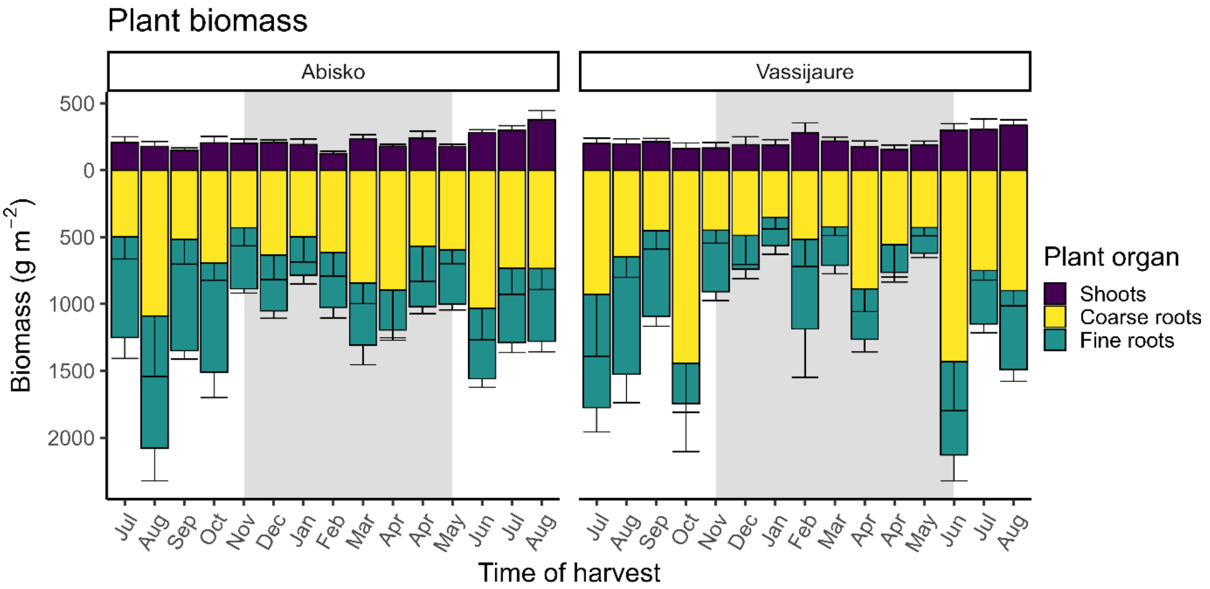

Supplementary Fig. S4: Year-round seasonal patterns of plant biomass (dry weight) separated into aboveground (S) and belowground (coarse and fine roots) parts in Abisko (left) and Vassijaure (right). Note that there is an early and a late April. Shoots include leaves and branches; coarse roots are roots > 2 mm in diameter, while fine roots are roots < 2 mm in diameter. The grey shading in the background indicates the snow-covered period. Monthly bars refer to when the experimental plots were harvested and represent estimated uptake over the preceding three-week period between labelling and harvest. Error bars represent standard errors (n = 5).

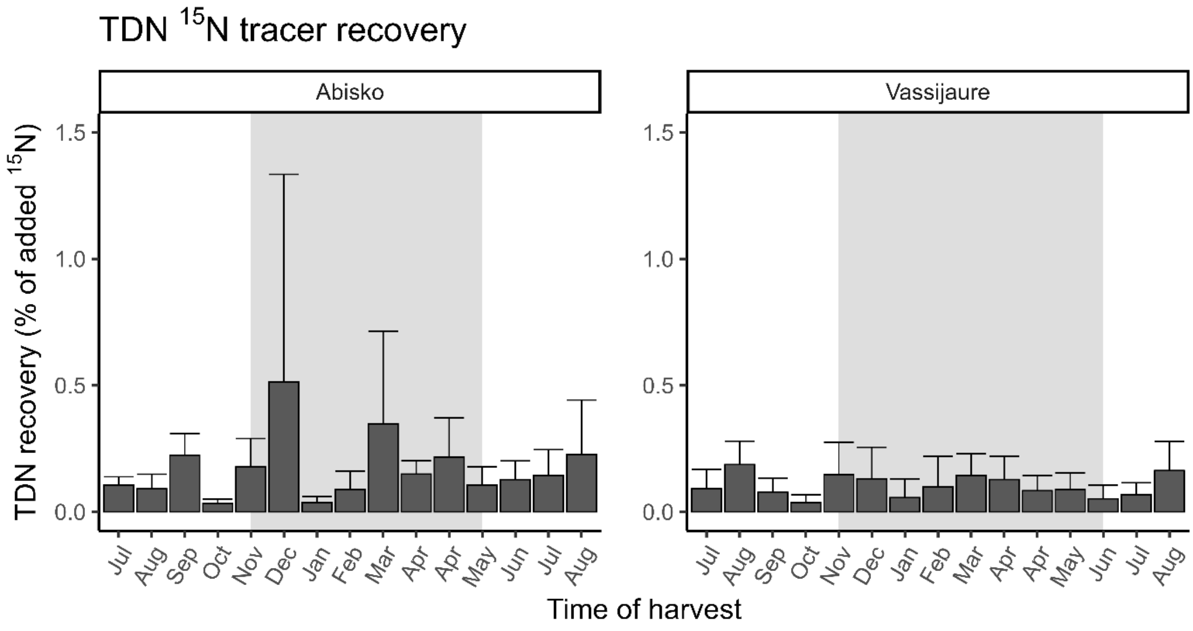

Supplementary Fig. S5: Year-round seasonal patterns of total dissolved nitrogen (TDN) (absolute) recovery of ^{15}N tracer (% of addition), in Abisko (left) and Vassijaure (right). Note that there is an early and a late April. The grey shading in the background indicates the snow-covered period. Monthly bars refer to when the experimental plots were harvested and represent estimated uptake over the preceding three-week period between labelling and harvest. Error bars represent 95% confidence intervals (n = 5).

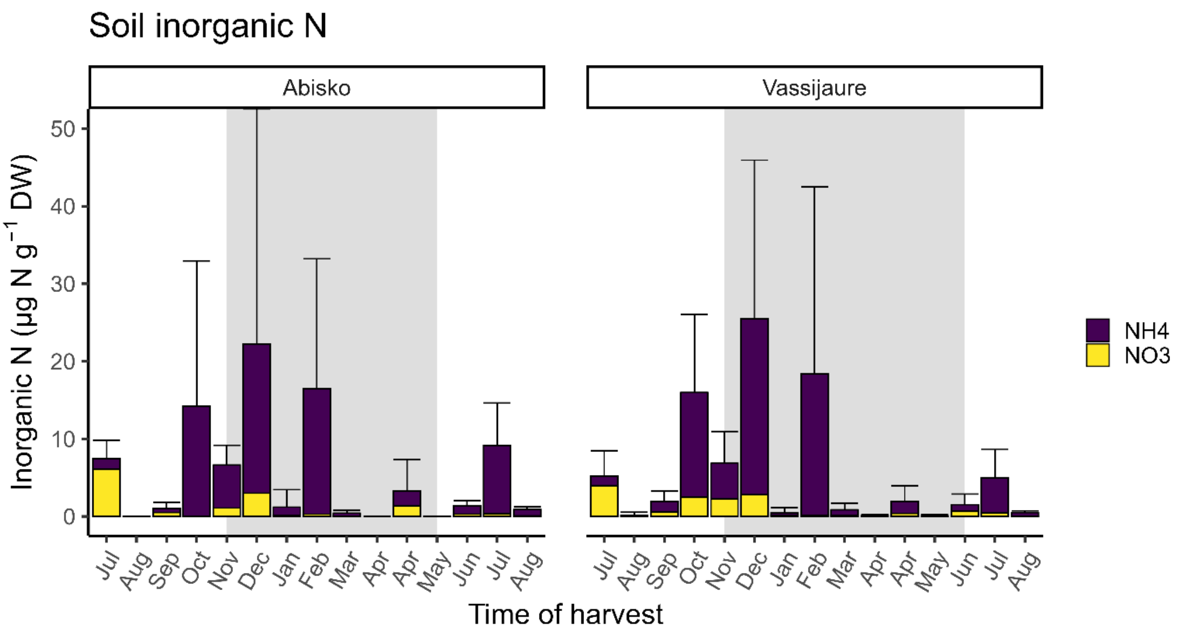

Supplementary Fig. S6: Year-round seasonal patterns of extracted soil inorganic N per g dry weight (DW) at harvest, in soils at Abisko (left) and Vassijaure (right) split between ammonium (NH_4) and nitrate (NO_3). Note that there is an early and a late April harvest. The grey shading in the background indicates the snow-covered period as measured on the plots. Error bars represent 95% confidence intervals (n = 5).

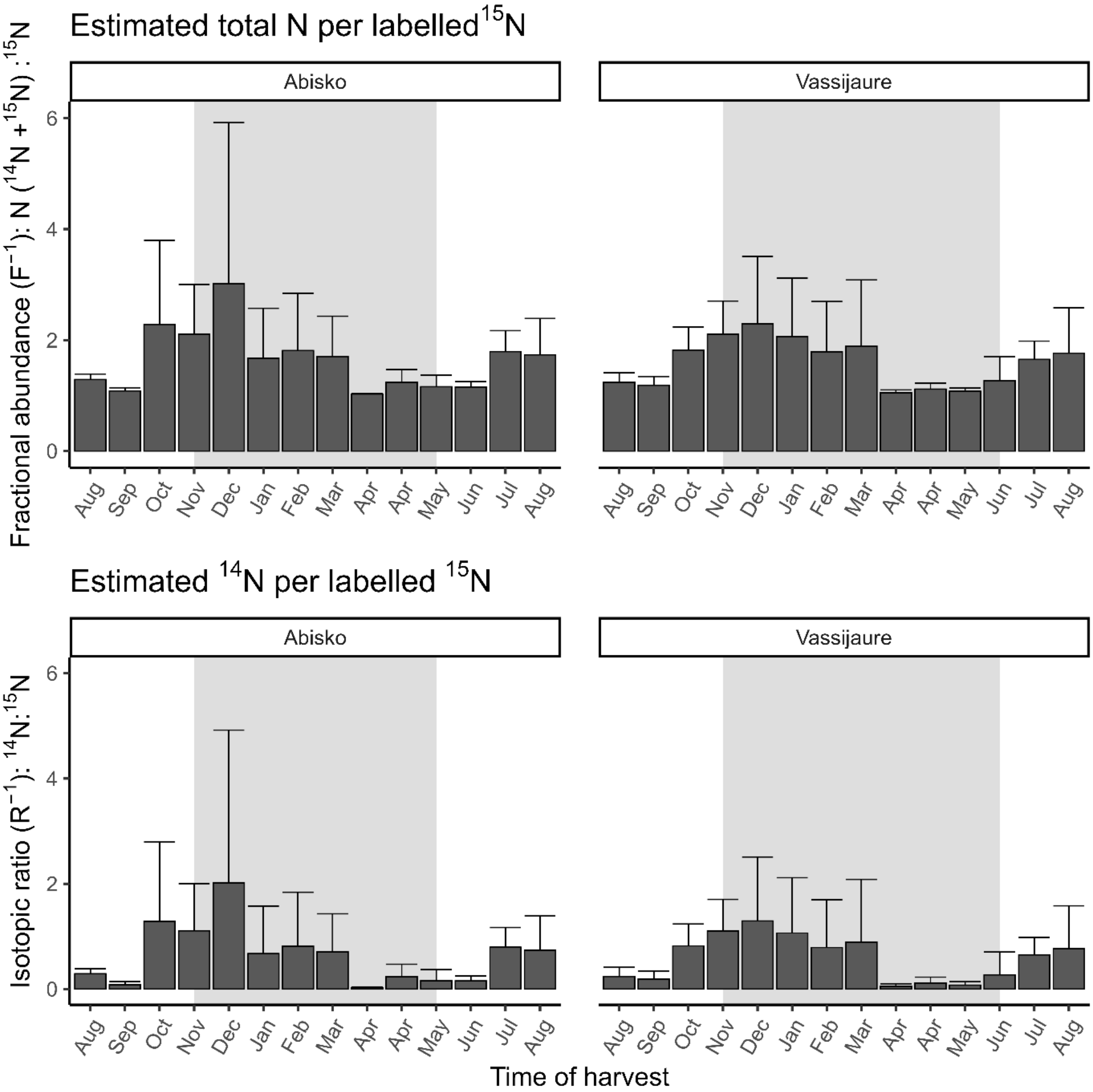

Supplementary Fig. S7: Year-round seasonal patterns of estimated inverse fractional abundance (F^{-1}; upper graphs) and isotopic ratio (R^{-1}; lower graphs) of ^{15}N in the soil inorganic N-pool during uptake, as an average over the three-week labelling period; in Abisko (left) and Vassijaure (right). Note that there is an early and a late April. The grey shading in the background indicates the snow-covered period. Monthly bars refer to when the experimental plots were harvested and represent 'high' enrichment estimated averages over the preceding three-week period between labelling and harvest. The high and low estimates refer to estimated enrichment (15N) of the soil inorganic N pool (see Suppl. Methods for how the estimates were made and Table S2 for all values). Error bars represent 95% confidence intervals (n = 5).

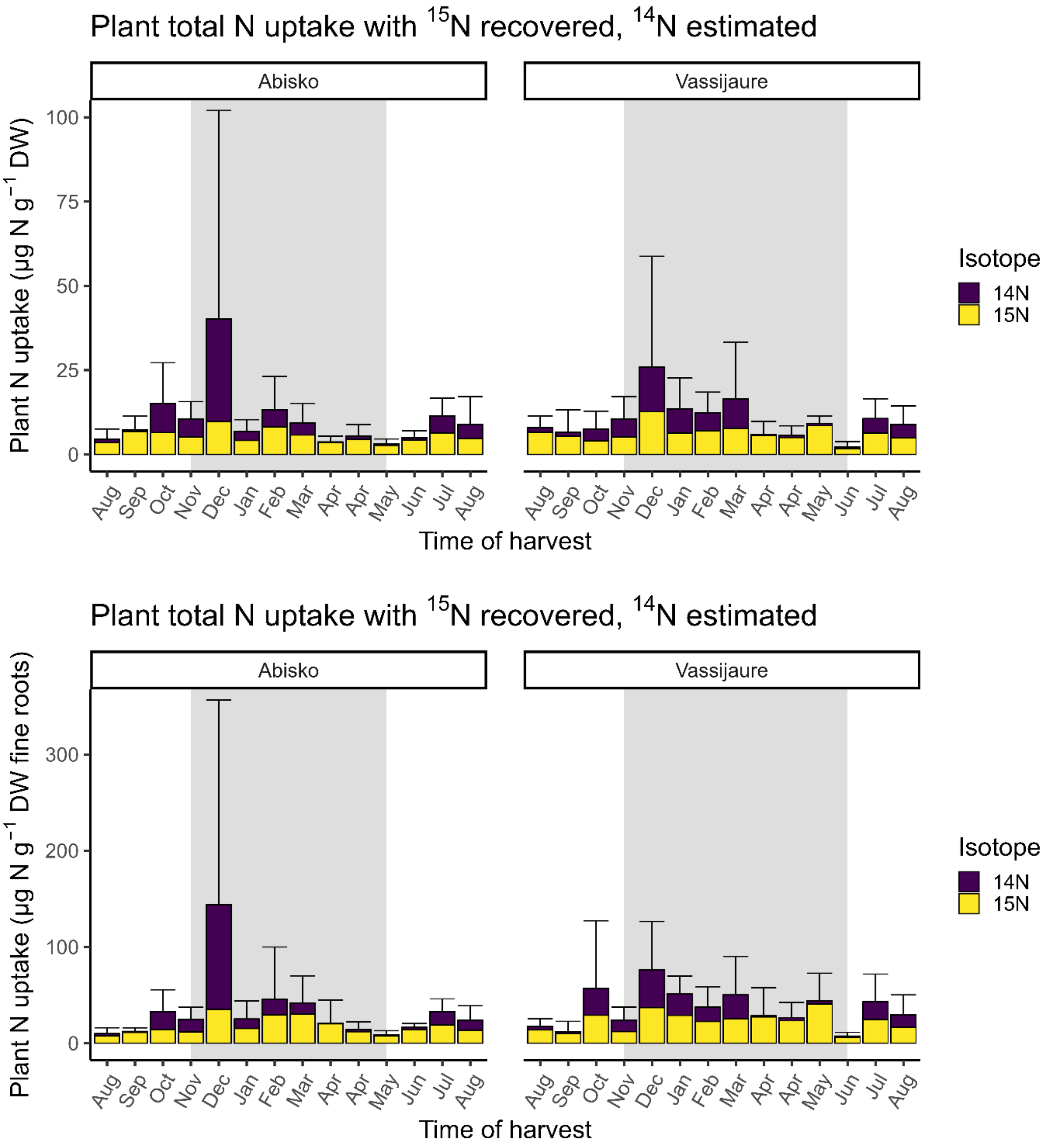

Supplementary Fig. S8: Year-round seasonal patterns of estimated plant total N-uptake in Abisko (left) and Vassijaure (right), 2019–2020, expressed per total plant dry weight (DW, upper panels) or per fine root DW (lower panels). The total N-uptake is the combined uptake of ^{14}N + ^{15}N, where the ^{15}N is from measured recovery after ^{15}N-labelling, and ^{14}N is estimated based on ^{15}N-recovery and the average fractional abundance of ^{15}N in the dissolved inorganic nitrogen pool (see Suppl. Methods). Note that there is an early and a late April measurement. The grey shading in the background indicates the snow-covered period. Monthly bars refer to when the experimental plots were harvested and represent estimated uptake over the preceding three-week period between labelling and harvest. Error bars represent 95% confidence intervals (n = 5). Significant effects (rmANOVA with a priori contrasts of seasons): Plant total N-uptake per total plant DW: MP $\chi^2 = 79.6$, df = 13, $p < 0.001$, Site × MP $\chi^2 = 23.4$, df = 13, $p = 0.037$, Abisko: contrast 3: t = 5.6, df = 52, $p < 0.001$, Vassijaure: contrast 3: t = 4.8, df = 52, $p < 0.001$. Plant total N-uptake per fine-root DW: MP $\chi^2 = 84.8$, df = 13, $p < 0.001$, Site × MP $\chi^2 = 22.9$, df = 13, $p = 0.042$, Abisko: contrast 3: t = 4.9, df = 52, $p < 0.001$, Vassijaure: contrast 2: t =

2.4, df =52, p = 0.022, contrast 3: t = 3.4, df = 52, $p = 0.001$. See Fig. 1 and Table S3 for details on definition of season contrasts.

References

Hayes JM. **2004**. An Introduction to Isotopic Calculations. Woods Hole Oceanographic Institution, MA, USA
